# Characterization of the *Plasmodium falciparum* and *P. berghei* glycerol 3‐phosphate acyltransferase involved in FASII fatty acid utilization in the malaria parasite apicoplast

**DOI:** 10.1111/cmi.12633

**Published:** 2016-08-01

**Authors:** Melanie J. Shears, James I. MacRae, Vanessa Mollard, Christopher D. Goodman, Angelika Sturm, Lindsey M. Orchard, Manuel Llinás, Malcolm J. McConville, Cyrille Y. Botté, Geoffrey I. McFadden

**Affiliations:** ^1^ School of BioSciencesUniversity of MelbourneVIC 3010Australia; ^2^ Department of Molecular Microbiology and ImmunologyJohns Hopkins University Bloomberg School of Public HealthBaltimoreMD21205United States; ^3^ The Francis Crick InstituteMetabolomicsThe Ridgeway, Mill HillLondonNW7 1AAUnited Kingdom; ^4^ Department of Biochemistry and Molecular Biology, Department of Chemistry and Center for Malaria ResearchPennsylvania State UniversityState CollegeUniversity ParkPA16802United States; ^5^ Department of Biochemistry and Molecular BiologyUniversity of MelbourneMelbourneVIC 3010Australia; ^6^ Apicolipid team, Institute for Advanced Biosciences UMR CNRS5309 INSMERM U1209Université Grenoble AlpesGrenobleFrance

## Abstract

Malaria parasites can synthesize fatty acids via a type II fatty acid synthesis (FASII) pathway located in their apicoplast. The FASII pathway has been pursued as an anti‐malarial drug target, but surprisingly little is known about its role in lipid metabolism. Here we characterize the apicoplast glycerol 3‐phosphate acyltransferase that acts immediately downstream of FASII in human (*Plasmodium falciparum*) and rodent (*Plasmodium berghei*) malaria parasites and investigate how this enzyme contributes to incorporating FASII fatty acids into precursors for membrane lipid synthesis. Apicoplast targeting of the *P. falciparum* and *P. berghei* enzymes are confirmed by fusion of the N‐terminal targeting sequence to GFP and 3′ tagging of the full length protein. Activity of the *P. falciparum* enzyme is demonstrated by complementation in mutant bacteria, and critical residues in the putative active site identified by site‐directed mutagenesis. Genetic disruption of the *P. falciparum* enzyme demonstrates it is dispensable in blood stage parasites, even in conditions known to induce FASII activity. Disruption of the *P. berghei* enzyme demonstrates it is dispensable in blood and mosquito stage parasites, and only essential for development in the late liver stage, consistent with the requirement for FASII in rodent malaria models. However, the *P. berghei* mutant liver stage phenotype is found to only partially phenocopy loss of FASII, suggesting newly made fatty acids can take multiple pathways out of the apicoplast and so giving new insight into the role of FASII and apicoplast glycerol 3‐phosphate acyltransferase in malaria parasites.

## Introduction

Malaria is caused by *Plasmodium* parasites, which have a complex life cycle involving multiple stages in the human and mosquito hosts (Greenwood *et al.*, 2008; Aly *et al.*, 2009). *Plasmodium* parasites require fatty acids for membrane lipid synthesis and other essential activities, and possess both an endogenous fatty acid synthesis pathway and mechanisms for scavenging fatty acids from the host (Déchamps *et al.*, 2010). The parasite fatty acid synthesis pathway is located in the apicoplast, a reduced plastid organelle homologous to chloroplasts of plants and algae (van Dooren and Striepen, 2013). As in chloroplasts and bacteria, the apicoplast harbours a dissociative, or type II fatty acid synthesis (FASII) pathway, which is intrinsically different to the FASI pathway of humans (Waller *et al.*, 1998). This characteristic, and the assumption that FASII was essential in blood stage parasites, led to its promotion as a target for anti‐malarial drugs, and considerable research was directed toward identifying FASII inhibitors (Shears *et al.*, 2015). However, transcriptomic and genetic studies with the human malaria parasite *Plasmodium falciparum* and the rodent models *Plasmodium berghei* and *Plasmodium yoelii* subsequently overturned this assumption, with FASII enzymes found to be minimally expressed (Bozdech *et al.*, 2003; Le Roch *et al.*, 2003; Llinás, 2006) and ultimately dispensable in blood stage parasites (Yu *et al.*, 2008; Vaughan *et al.*, 2009). These data not only indicated that FASII was invalid as a therapeutic target, but also that fatty acid scavenging was instead primarily responsible for supporting lipid synthesis at this stage. In *P. berghei* and *P. yoelii*, FASII was found to be essential for parasite development in the late liver stage, suggesting it might still be targeted for malaria prophylaxis (Yu *et al.*, 2008; Vaughan *et al.*, 2009). In *P. falciparum*, however, FASII was unexpectedly discovered to be essential for sporozoite development, precluding analysis of mutants in the liver stage and crucial validation of the pathway as a prophylactic target (van Schaijk *et al.*, 2014). These unanticipated findings emphasized a need for further research into FASII, and raised questions about both the role of the pathway in parasite lipid metabolism and its actual potential as a target for malaria prevention.

Several hypotheses have been proposed to explain why FASII is essential at certain stages of the malaria parasite life cycle. FASII primarily produces the fatty acid myristate (C14:0) (Botté *et al.*, 2013), which is predicted to be used for phospholipid synthesis because these make up the majority of parasite membranes (Déchamps *et al.*, 2010). FASII also produces octanoate (C8:0), but as this seems to only be essential for synthesis of lipoic acid for the apicoplast pyruvate dehydrogenase, itself a FASII enzyme, this function appears to be solely self‐sustaining (Storm and Müller, 2012). Based on the observations that FASII null mutants arrest at replicative life stages and show reduced growth and organelle development in the preceding stage (Yu *et al.*, 2008; Vaughan *et al.*, 2009; Pei *et al.*, 2010; Butler *et al.*, 2011; Annoura *et al.*, 2012; Nagel *et al.*, 2013; van Schaijk *et al.*, 2014), it has been proposed that FASII is required to supplement fatty acid scavenging and boost bulk membrane lipid synthesis (Yu *et al.*, 2008). Alternatively, it has been hypothesized that FASII is required for synthesis of certain essential lipids that cannot be acquired from the host (Tarun *et al.*, 2009). As FASII null mutants in rodent models consistently show reduced expression of the liver stage marker merozoite surface protein 1 (MSP1), it was suggested that FASII may be needed for production of the glycophosphatidylinositol (GPI) anchor of the protein (Tarun *et al.*, 2009). This in turn sparked the hypothesis that *P. falciparum* may be reliant on FASII in the mosquito stages for synthesis of the GPI anchor of the circumsporozoite protein (CSP) or other essential surface proteins (van Schaijk *et al.*, 2014), but so far there is no definitive experimental evidence to support or distinguish these hypotheses.

As in other organisms, malaria parasites are predicted to synthesize phospholipids and the lipid moieties of GPI anchors from a precursor known as phosphatidic acid (Déchamps *et al.*, 2010). Phosphatidic acid is produced via a two‐step pathway involving a glycerol 3‐phosphate acyltransferase (G3PAT) and lysophosphatidic acid acyltransferase (LPAAT), which rely on conserved histidine and aspartate residues in a ‘HX_4_D’ motif (Heath and Rock, 1998) to catalyse the attachment of fatty acids to the first and second positions of glycerol 3‐phosphate, respectively. *Plasmodium* parasites have been predicted to have two sets of these enzymes, one set in the apicoplast and another set in the endoplasmic reticulum (ER) (Santiago *et al.*, 2004; Lindner *et al.*, 2014). The identification of a putative mechanism for apicoplast fatty acid export (Ralph *et al.*, 2004) indicates FASII can potentially contribute fatty acids to both of these pathways. To date, however, there have been relatively few studies to provide evidence for FASII fatty acids being used in phosphatidic acid synthesis in either organelle. The *P. falciparum* ER G3PAT has been characterized and uses a range of fatty acids including myristate (C14:0) (Santiago *et al.*, 2004), providing suggestive data that FASII may provide substrates to this enzyme. The *P. yoelii* apicoplast G3PAT has also been characterized and its deletion found to phenocopy FASII null mutants in the late liver stage (Lindner *et al.*, 2014), providing a more tangible link between the two pathways in this model. Surprisingly, however, the same study also found the predicted *P. yoelii* apicoplast LPAAT was localized to the ER, indicating the apicoplast pathway may differ from that in other plastid‐bearing organisms and instead only produce the intermediate lysophosphatidic acid (Lindner *et al.*, 2014). Therefore, although the apicoplast and ER acyltransferases are predicted to be integral for linking FASII with membrane lipid synthesis, their precise role in FASII fatty acid metabolism is yet to be elucidated.

In this study, we characterize the apicoplast G3PAT of *P. falciparum* (*Pf* apiG3PAT) and *P. berghei* (*Pb* apiG3PAT) to further explore how this enzyme contributes to FASII fatty acid metabolism. We confirm the apicoplast targeting and activity of *Pf* apiG3PAT and investigate residues in its predicted active site by targeted mutagenesis. We establish the dispensability of *Pf* apiG3PAT in the blood stage, and compare the phenotype of mutant and wild type parasites in both standard media and conditions that induce FASII activity to make additional inferences about fatty acid metabolism at this stage. Performing complementary studies in *P. berghei*, we verify the apicoplast localization of *Pb* apiG3PAT and demonstrate the enzyme is critical for parasite development in the late liver stage, consistent with findings for its *P. yoelii* homolog. We further show that the *Pb* apiG3PAT deletion mutant phenocopies the *P. berghei* FASII null mutant phenotype in many respects, but not in the severity of the MSP1 expression defect, revealing novel insight into the role of the enzyme in lipid metabolism and the complex interplay between pathways for fatty acid metabolism in the apicoplast and ER**.**

## Results and discussion

### Pf *apiG3PAT is targeted to the apicoplast and functions as a typical G3PAT*

**Figure 1:**
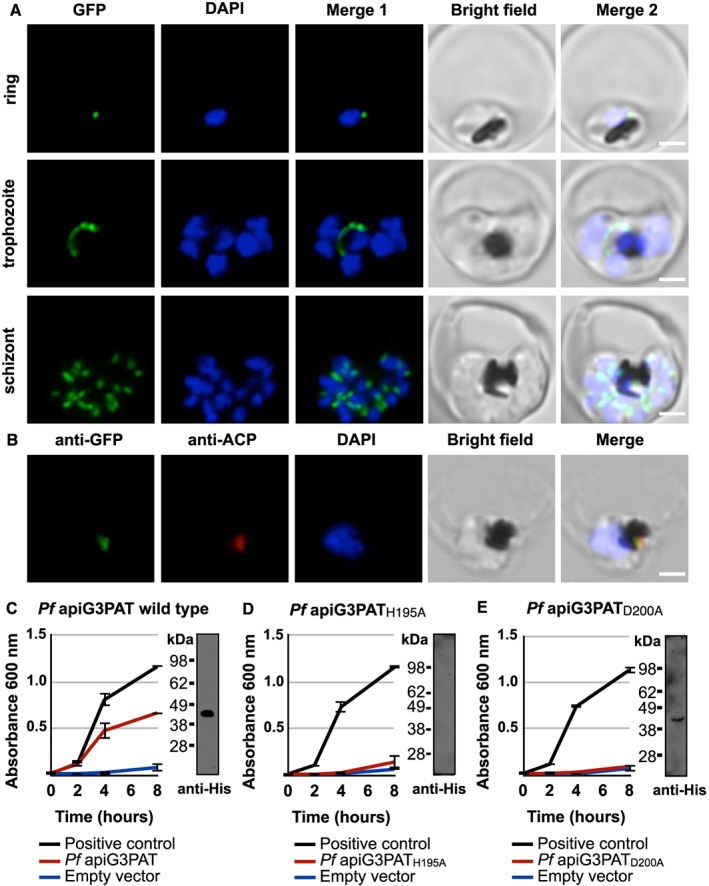
*Pf* apiG3PAT has a functional apicoplast targeting sequence and rescues growth in a G3PAT‐deficient mutant strain of *E. coli*. A. Live fluorescence microscopy of *Pf apiG3PAT_1–70_ gfp* parasites shows the predicted *Pf* apiG3PAT apicoplast targeting sequence directs GFP to a discrete cellular compartment in rings, trophozoites and schizonts. B. Immunofluorescence microscopy of *Pf apiG3PAT_1–70_ gfp* parasites using antibodies against GFP and the apicoplast marker ACP demonstrates the predicted *Pf* apiG3PAT apicoplast targeting sequence targets GFP to the apicoplast. DNA stained with DAPI. Scale 3 µm. C. *Pf* apiG3PAT partially restores growth in a G3PAT‐deficient mutant of *E. coli*, demonstrating it is active as a G3PAT. Bacteria were transformed with a vector encoding a His‐tagged version of *Pf* apiG3PAT, the empty vector or *E. coli* G3PAT positive control. Western blot of bacterial extracts confirms expression of the tagged *Pf* apiG3PAT (expected mass of 47 kDa). D–E. Mutation of the conserved histidine or aspartate in the ‘HX_4_D’ motif of *Pf* apiG3PAT abolishes its ability to restore growth in the *E. coli* mutant. Bacteria were transformed with vectors encoding His‐tagged versions of the histidine to alanine mutant (*Pf* apiG3PAT_H195A_), aspartate to alanine mutant (*Pf* apiG3PAT_D200A_), empty vector or *E. coli* G3PAT positive control. Western blot of bacterial extracts confirms expression of *Pf* apiG3PAT_D200A_, but fails to detect *Pf* apiG3PAT_H195A_, suggesting the conserved histidine may be required for correct folding or stability of the enzyme.

*Pf* apiG3PAT was initially implicated as a putative apicoplast protein on the basis of a predicted apicoplast targeting sequence at its N‐terminus (Ralph *et al.*, 2004). Further *in silico* analysis of *Pf* apiG3PAT confirmed the presence of a putative apicoplast targeting sequence of 70 amino acids that contained a predicted signal peptide and predicted transit peptide as expected (Zuegge *et al.*, 2001; Foth *et al.*, 2003; Petersen *et al.*, 2011) (Supplementary Fig. 1). To test if this putative apicoplast targeting sequence was functional, we generated the transgenic *Pf apiG3PAT_1–70_ gfp* parasite line, which expressed the predicted *Pf* apiG3PAT targeting sequence fused to a C‐terminal green fluorescent protein (GFP) reporter. Live fluorescence microscopy of blood stage *Pf apiG3PAT_1–70_ gfp* parasites revealed that GFP was directed to a discrete cellular compartment characteristic of the apicoplast (van Dooren *et al.*, 2005) in ring, trophozoite and schizont stage parasites (Fig. 1A). Immunofluorescence microscopy of *Pf apiG3PAT_1–70_ gfp* parasites confirmed GFP was targeted to the apicoplast by co‐localization with the apicoplast marker, acyl carrier protein (ACP; Waller *et al.*, 2000) (Fig. 1B). Thus, the predicted *Pf* apiG3PAT targeting sequence was sufficient to direct GFP into the apicoplast, consistent with *Pf* apiG3PAT being an apicoplast resident protein.

*Pf* apiG3PAT was identified as a putative G3PAT based on sequence similarity to known G3PATs (Ralph *et al.*, 2004) and the presence of the ‘HX_4_D’ catalytic motif characteristic of the enzyme class (Heath and Rock, 1998) (Supplementary Fig. 1). To test whether *Pf* apiG3PAT had the predicted activity, we employed an established complementation assay using the G3PAT‐deficient *plsB26* mutant strain of *Escherichia coli* (Bell, 1974). The *plsB26* mutant has a defective G3PAT that renders it unable to grow in minimal media lacking glycerol unless complemented with a functional enzyme (Bell, 1974). To test *Pf* apiG3PAT for activity, we transformed mutant bacteria with a vector encoding a hexahistidine‐tagged version of the protein (without the apicoplast targeting sequence), then monitored for growth on minimal media without glycerol in two separate experiments. As controls, we transformed bacteria with the empty vector or a positive control vector encoding a His‐tagged version of the wild type *E. coli* G3PAT. Bacteria transformed with the *Pf* apiG3PAT vector grew markedly better than the empty vector control, and achieved approximately 60% of the growth observed for the positive control (Fig. 1C), consistent with findings for *Py* apiG3PAT (Lindner *et al.*, 2014). Western blot analysis of extracts from transformed bacteria using anti‐His antibodies also verified that the tagged *Pf* apiG3PAT was expressed and soluble as expected. This demonstrated that *Pf* apiG3PAT expression was sufficient to partially restore growth in the G3PAT‐deficient bacterial mutant, confirming that *Pf* apiG3PAT was active as a G3PAT.

Having demonstrated that *Pf* apiG3PAT was active, we next sought to test whether, as in other characterized G3PATs (Heath and Rock, 1998; Lewin *et al.*, 1999; Turnbull *et al.*, 2001; Tamada *et al.*, 2004), the conserved histidine and aspartate residues of the ‘HX_4_D’ motif were important for catalysis. To investigate this, we individually mutated the conserved histidine and aspartate residues of *Pf* apiG3PAT to alanine, then tested the resultant proteins for their ability to rescue growth in the bacterial complementation assay. Bacteria transformed with the vector encoding the *Pf* apiG3PAT_H195A_ histidine to alanine mutant had the same residual growth rate as bacteria transfected with the empty vector control, suggesting the conserved histidine was required for *Pf* apiG3PAT activity (Fig. 1D). However, we were unable to detect this protein by Western blot analysis despite numerous attempts, and so could not exclude the alternate hypothesis that alteration of the residue had affected the expression, folding or stability of the protein. Bacteria transformed with the vector encoding the *Pf* apiG3PAT_D200A_ aspartate to alanine mutant were similarly indistinguishable from the empty vector control, and in this case weak expression of the protein could be detected by Western blot analysis (Fig. 1E). These observations indicated that *Pf* apiG3PAT likely resembled characterized G3PATs in its reliance on the conserved histidine and aspartate of the ‘HX_4_D’ motif for activity or folding, indicating the enzymes putatively share a common mechanism of catalysis.

### Pf *apiG3PAT is dispensable in asexual blood stage parasites*

Having confirmed the apicoplast localization and activity of *Pf* apiG3PAT, we next sought to determine the requirement for the enzyme in blood stage parasites. Because we expected *Pf* apiG3PAT would primarily or exclusively use FASII fatty acids, we predicted that it would be dispensable in asexual blood stage parasites. To test this we used a double cross over homologous recombination strategy to disrupt the *Pf* apiG3PAT locus, inserting the human dihydrofolate reductase (hDHFR) drug resistance cassette into coding sequence to truncate the enzyme and eliminate the ‘HX_4_D’ active site motif (Supplementary Fig. 2). Drug‐resistant parasites were obtained from two independent transfections in the D10 wild type background, and the successful integration of the hDHFR cassette into the *Pf* apiG3PAT locus was confirmed by PCR for each line (Supplementary Fig. 2). We also attempted to transfect into the mosquito‐transmissible NF54 wild type background on three separate occasions without success (data not shown). Proceeding with the two lines in the D10 background, we observed both *Pf apiG3PAT (−)* lines were viable and grew normally as determined by frequency of subculturing, suggesting *Pf* apiG3PAT was indeed dispensable for blood stage growth.

As we had employed a transfection strategy that relied on negative selection to eliminate wild type parasites rather than cloning by limiting dilution (Maier *et al.*, 2006), we also sought to verify the disruption of the *Pf* apiG3PAT locus by whole genome sequencing of one line. This analysis again confirmed the successful integration of the hDHFR cassette into the *Pf* apiG3PAT locus, and although three single base changes were identified between the mutant line and the wild type parent across the entire genome, targeted PCR‐based sequencing of these regions in the second *Pf apiG3PAT (−)* line revealed that they were not conserved (sequencing data deposited in NCBI Short Read Archive under accession number SRP071808, PCR data not shown). Therefore, we reasoned the ability to recover *Pf apiG3PAT (−)* parasites was not because of compensatory mutations or insertion of the selectable marker at other loci, and hence that *Pf* apiG3PAT activity was truly dispensable in blood stage parasites.

**Figure 2:**
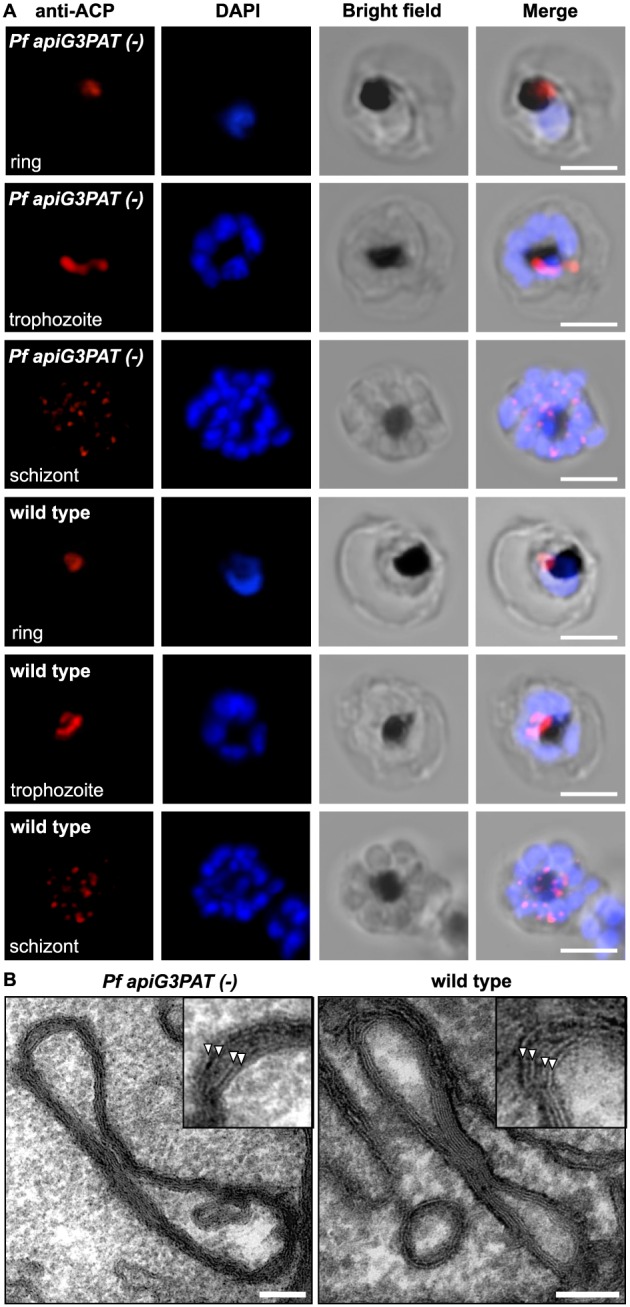
*Pf apiG3PAT (−)* parasites have normal apicoplast morphology and development in the blood stage. A. Immunofluorescence microscopy of *Pf apiG3PAT (−)* and wild type parasites using antibodies against the apicoplast marker ACP demonstrates disruption of the enzyme has no effect apicoplast morphology in rings, trophozoites or schizonts. DNA stained with DAPI. Scale 3 µm. B. Transmission electron microscopy of trophozoite stage *Pf apiG3PAT (−)* and wild type parasites confirms disruption of the enzyme has no effect on apicoplast structure or membrane organization. Magnified images (inset) show the four apicoplast membranes are arranged in pairs (arrowheads) in both lines. Scale 100 nm.

To investigate whether disruption of *Pf* apiG3PAT produced any subtle phenotype in the growth or development of blood stage parasites, one *Pf apiG3PAT (−)* line was selected for further characterization. First, to monitor apicoplast development in the asexual blood stage cycle, we performed immunofluorescence microscopy using antibodies against the apicoplast marker ACP (Waller *et al.*, 2000). No difference was observed between the *Pf apiG3PAT (−)* line and wild type parasites in apicoplast appearance in ring, trophozoite or schizont stages, indicating disruption of the enzyme did not impact apicoplast development in the blood stage (Fig. 2A). Transmission electron microscopy further confirmed this finding, with typical apicoplast ultrastructure and membrane organization observed in trophozoite stage parasites of both lines, and whorls of membrane seen in the apicoplast lumen in the *Pf apiG3PAT (−)* parasite as previously reported for wild type (Lemgruber *et al.*, 2013) (Fig. 2B). Disruption of *Pf* apiG3PAT therefore had no appreciable impact on apicoplast development in blood stage parasites grown in standard culture conditions, indicating the enzyme was not required for apicoplast membrane synthesis and was either non‐active or functionally redundant in this stage.

**Figure 3:**
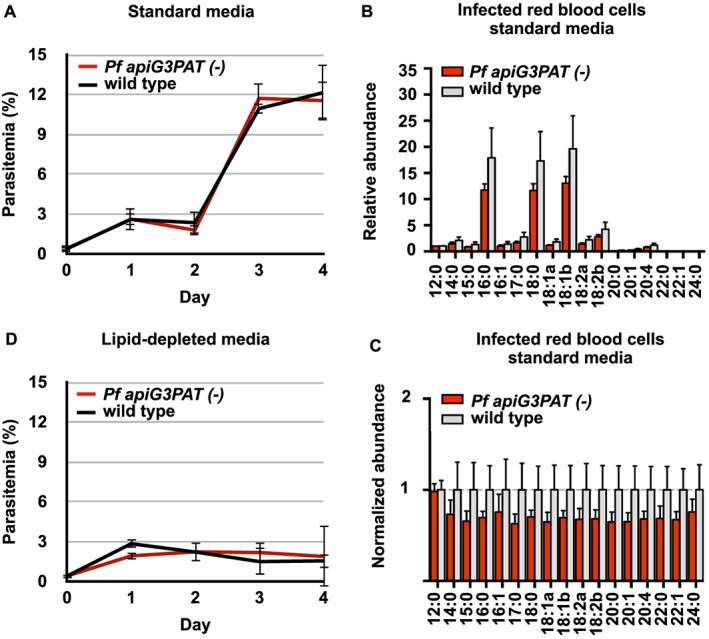
*Pf apiG3PAT (−)* and wild type parasites show similar growth on standard and lipid‐depleted media and have comparable lipid fatty acid profiles. A. *Pf apiG3PAT (−)* and wild type parasites show similar replication rates in standard media in a four‐day blood stage growth assay. Error bars show mean of three biological replicates ± standard deviation. B and C. Relative and normalized fatty acid abundance in lipid extracts from magnetically isolated *Pf apiG3PAT (−)* and wild type infected red blood cells grown in standard media. Importantly, although the mean values were consistently lower in *Pf apiG3PAT (−)* samples, these differences were non‐significant as determined by a *t*‐test (*p*‐value >0.05). Error bars show mean of four biological replicates ± standard deviation. See Supplementary Table 2 for all values. D. *Pf apiG3PAT (−)* and wild type parasites show a similar decrease in replication rate when transferred to lipid‐depleted media containing only the minimal fatty acid supplementation required for growth, suggesting the enzyme is not required for growth in these conditions.

To investigate whether disruption of *Pf* apiG3PAT had any effect on parasite growth or cell cycle progression, the replication rate of synchronized parasites from the *Pf apiG3PAT (−)* and wild type line were compared using an established four day growth assay (Mitamura *et al.*, 2000; Mi‐Ichi *et al.*, 2006). No difference was observed between *Pf apiG3PAT (−)* and wild type parasites in replication rate in standard culture conditions, with both lines found to increase in parasitemia between five and six fold per cycle (Fig. 3A). Similarly, no appreciable difference was detected between *Pf apiG3PAT (−)* and wild type parasites in the proportion of rings or trophozoites present each day of the assay (Supplementary Fig. 3), providing preliminary evidence that disruption of *Pf* apiG3PAT had no effect on either parasite growth or cell progression in the blood stage. This lack of growth phenotype was further supported by GC‐MS analysis of magnetically isolated *Pf apiG3PAT (−)* and wild type infected red blood cells, which showed disruption of the enzyme had no significant affect on the overall fatty acid profile of parasite lipids (Fig. 3B and C, Supplementary Table 2). Together, these findings demonstrated that *Pf* apiG3PAT was dispensable in blood stage parasites in standard culture conditions, and suggested the enzyme did not normally contribute to bulk membrane lipid synthesis in at this stage.

Last, as it has previously been reported that *Pf* apiG3PAT and FASII expression is up‐regulated in *P. falciparum ex vivo* isolates displaying a ‘starvation’ transcriptional response (Daily *et al.*, 2007), we hypothesized that *Pf* apiG3PAT may be important in blood stage parasites in environments where exogenous fatty acids are limiting. To test this, we repeated the growth assay using lipid‐depleted media containing only palmitate (C16:0) and oleate (C18:1), because these are the minimum fatty acid set required to support parasite growth, and the conditions were previously shown to also induce FASII activity (Botté *et al.*, 2013). Again, no difference was observed between the *Pf apiG3PAT (−)* line and wild type in their fold replication per cycle in lipid‐depleted conditions, with both lines failing to increase in parasitemia after the first cycle (Fig. 3D). Furthermore, although the growth of *Pf apiG3PAT (−)* and wild type parasites was clearly reduced in lipid‐depleted media, both displayed normal cell cycle progression, indicating a low number of successful replication events had indeed taken place (Supplementary Fig. 3). Thus disruption of *Pf* apiG3PAT caused no further reduction in growth in lipid‐depleted media beyond that observed for wild type. This suggested that incorporation of FASII fatty acids into membrane lipid precursors in the apicoplast was not essential at this stage, and by extension, that these fatty acids were instead likely exported and utilized by other pathways.

### Pb *apiG3PAT is targeted to the apicoplast and expressed in liver stage parasites*

**Figure 4:**
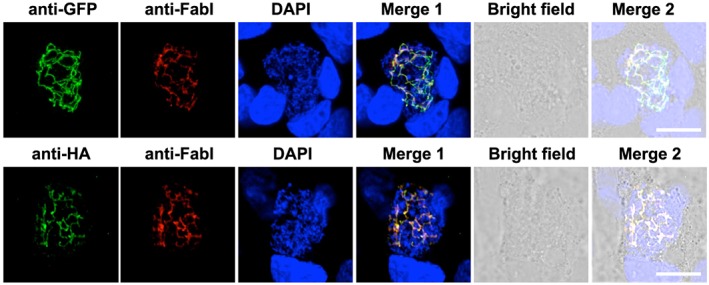
*Pb* apiG3PAT is expressed in liver stage parasites and localizes to the apicoplast. Immunofluorescence microscopy of *Pb apiG3PAT ha gfp* liver stage parasites 48 h post‐infection confirms the GFP and HA signals co‐localize with the apicoplast marker FabI. Parasite and host cell nuclei stained with DAPI. Scale 10 µm.

*Pb* apiG3PAT shares strong sequence similarity with *Pf* apiG3PAT and is likewise predicted to possess an apicoplast targeting sequence at its N‐terminus (Supplementary Fig. 1). To verify that *Pb* apiG3PAT is targeted to the apicoplast, we used a 3′ gene replacement strategy to generate the *Pb apiG3PAT ha gfp* transgenic line, which expressed the full‐length protein fused to C‐terminal hemagglutinin (HA) and GFP tags (Supplementary Fig. 4). We chose this approach as it resulted in the tagged protein being expressed from the endogenous promoter, and could therefore also provide information about when in the life cycle *Pb* apiG3PAT was expressed. No GFP fluorescence was observed in blood stage parasites, oocysts or salivary gland sporozoites by live fluorescence microscopy, indicating *Pb* apiG3PAT was not expressed at detectable levels in these stages (data not shown). GFP fluorescence was however observed in liver stage parasites in a structure resembling the apicoplast (Stanway *et al.*, 2009), indicating that *Pb* apiG3PAT is normally expressed at this stage (Supplementary Fig. 5). Apicoplast targeting of *Pb* apiG3PAT was then confirmed by immunofluorescence microscopy and colocalization of the HA and GFP tags with the FASII enzyme FabI (Yu *et al.*, 2008) (Fig. 4). This demonstrated that *Pb* apiG3PAT is localized to the apicoplast and expressed in liver stage parasites, consistent with the observed up‐regulation of FASII enzymes at this stage in rodent malaria models (Tarun *et al.*, 2008).

### Pb *apiG3PAT is dispensable in blood and mosquito stage parasites*

**Figure 5:**
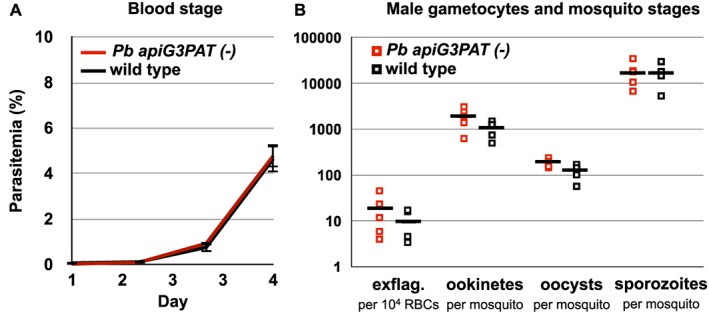
*Pb apiG3PAT (−)* parasites show normal development in the blood and mosquito stages. A. Blood stage growth of *Pb apiG3PAT (−)* and wild type parasites is indistinguishable in a four‐day *in vivo* growth assay. Error bars show mean of six mice ± standard error. B. Numbers of exflagellating male gametocytes and mosquito stage parasites in the *Pb apiG3PAT (−)* and wild type lines are comparable. Exflagellation rate determined per 10^4^ red blood cells (RBCs) immediately prior to mosquito infection, and ookinetes, oocysts and salivary gland sporozoites counted 20–22 h, 14–15 days and 21–22 days post‐infection, respectively. Results from five biological replicates, with mean values indicated by bars.

Having shown that *Pb* apiG3PAT was targeted to the apicoplast and expressed in liver stage parasites, we next sought to test whether it was essential for late liver stage development like *Py* apiG3PAT (Lindner *et al.*, 2014) and FASII (Yu *et al.*, 2008; Vaughan *et al.*, 2009) in rodent malaria models. For this, a double cross over homologous recombination strategy was used to disrupt *Pb* apiG3PAT by inserting the hDHFR cassette into its coding sequence, mirroring the approach we used to disrupt *Pf* apiG3PAT (Supplementary Fig. 6). Drug‐resistant parasites were obtained from two independent transfections and cloned by limiting dilution, and successful disruption of the *Pb* apiG3PAT locus in each clone was confirmed by PCR (Supplementary Fig. 6). The ability to recover *Pb apiG3PAT (−)* parasites indicated the enzyme was not essential in asexual blood stage parasites, consistent with the dispensability of *Py* apiG3PAT (Lindner *et al.*, 2014) and FASII at this stage (Yu *et al.*, 2008; Vaughan *et al.*, 2009). Furthermore, comparison of the *Pb apiG3PAT (−)* clone 1 to wild type parasites in an *in vivo* blood stage growth assay revealed no apparent difference between the lines (Fig. 5A), confirming that as for *Pf* apiG3PAT, disruption of the *P. berghei* enzyme had no appreciable impact on growth at this stage.

Next, to determine whether disruption of *Pb* apiG3PAT altered parasite transmission or development in the mosquito, we compared the numbers of male gametocytes, ookinetes, oocysts and salivary gland sporozoites produced by *Pb apiG3PAT (−)* and wild type parasites. Preliminary experiments indicated that neither of the *Pb apiG3PAT (−)* clones displayed any defects in mosquito stage development, consistent with our inability to detect expression of *Pb* apiG3PAT by fluorescence microscopy at these stages. More detailed analysis of *Pb apiG3PAT (−)* clone 1 further confirmed that disruption of the enzyme had no appreciable impact on these stages, with the mean numbers of male gametocytes, ookinetes, oocysts or salivary gland sporozoites from five experiments closely approximating those observed for wild type (Fig. 5B). Disruption of *Pb* apiG3PAT therefore had no apparent impact on parasite transmission or development in the mosquito, consistent with the finding that neither *Py* apiG3PAT (Lindner *et al.*, 2014) or FASII (Yu *et al.*, 2008; Vaughan *et al.*, 2009) is required in these life stages in rodent malaria models.

### Pb *apiG3PAT is required for normal sporozoite infectivity* in vivo

After establishing that disruption of *Pb* apiG3PAT did not affect mosquito stage development, we sought to test whether *Pb apiG3PAT (−)* sporozoites differed from wild type in their ability to produce patent blood stage infections *in vivo*. To compare the infectivity of *Pb apiG3PAT (−)* and wild type, we intravenously injected sporozoites into outbred Swiss Webster mice, and time to blood stage patency was monitored. Preliminary experiments with the *Pb apiG3PAT (−)* clones indicated they were both markedly attenuated in infectivity relative to wild type (data not shown), consistent with the observed phenotype of the *P. berghei* FASII null mutants (Yu *et al.*, 2008; Annoura *et al.*, 2012; Nagel *et al.*, 2013). Selecting *Pb apiG3PAT (−)* clone 1 for further analysis, we then compared time to patency with wild type after injection of 1000 or 10 000 sporozoites in six independent experiments. Similar to previous experiments with outbred mice (Jaffe *et al.*, 1990; Scheller *et al.*, 1994), injection of 10 000 wild type sporozoites produced infections in all six mice after an average of 4.2 days, while injection of 1000 wild type sporozoites produced infections in four mice after an average of 5.8 days (Table 1). By comparison, injection of either dose of *Pb apiG3PAT (−)* sporozoites produced infections in a smaller fraction of mice and with substantial delays (4–5 days) relative to wild type (Table 1). To test whether the attenuation of *Pb apiG3PAT (−)* blood stage patency was influenced by the route of sporozoite infection, we also assessed the time to patency after infection by biting with 10 mosquitoes. Once again, *Pb apiG3PAT (−)* sporozoites produced infections in fewer mice than wild type, with the one mouse that did become infected doing so with an approximate 6 day delay relative to the control (Table 1). This demonstrated that *Pb apiG3PAT (−)* sporozoites were markedly less infective than wild type regardless of the infection route, with disruption of the enzyme resulting in comparable attenuation to that reported for the *P. berghei* FASII null mutants (Yu *et al.*, 2008; Annoura *et al.*, 2012; Nagel *et al.*, 2013).

**Table 1:** *Pb apiG3PAT (−)* parasites show reduced sporozoite infectivity *in vivo.*

Strain	Route	Sporozoites	Fraction patent	Days to patency^a^
*Pb apiG3PAT (−)*	Intravenous	10 000	5/6	8.8
Wild type	Intravenous	10 000	6/6	4.2
*Pb apiG3PAT (−)*	Intravenous	1000	1/6	10
*Pb* ANKA WT	Intravenous	1000	4/6	5.8
*Pb apiG3PAT (−)*	Mosquito bite	10 mosquitoes	1/6	11
*Pb* ANKA WT	Mosquito bite	10 mosquitoes	6/6	4.5

a Mean days to patency calculated from mice that developed a patent infection.

### Pb *apiG3PAT is required for the normal growth and maturation of late liver stage parasites* in vitro

**Figure 6:**
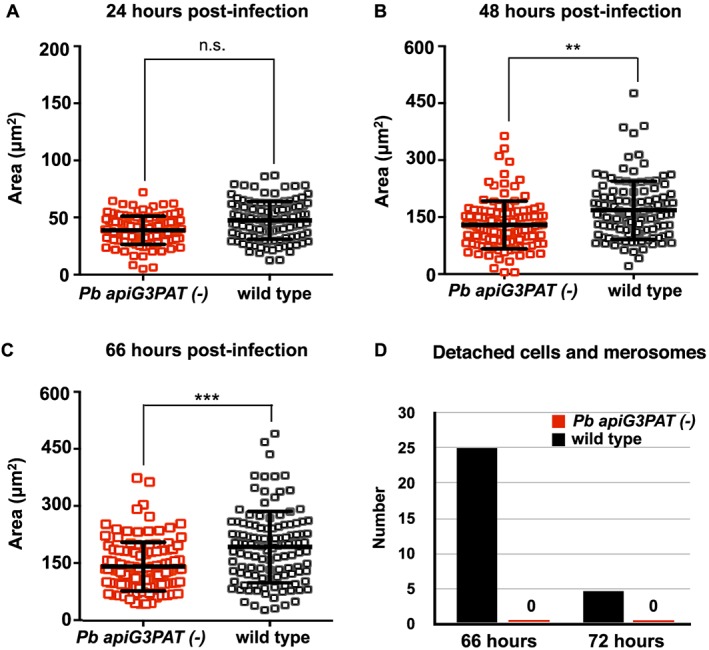
*Pb apiG3PAT (−)* parasites are smaller in the late liver stage and fail to produce merosomes *in vitro*. A. Cross‐sectional area of *Pb apiG3PAT (−)* and wild type liver stage parasites was determined by immunofluorescent labelling with antibodies against the cytosolic protein HSP70. *Pb apiG3PAT (−)* and wild type parasites do not significantly differ in size at 24 h post‐infection as determined by a two tailed *t*‐test (n.s., non‐significant). B and C. *Pb apiG3PAT (−)* parasites are significantly smaller than wild type at 48 and 66 h post‐infection, indicating the enzyme is required for normal parasite growth in the late liver stage (**, *p*‐value <0.05, ***, *p*‐value <0.001). Error bars show mean ± standard error. D. Numbers of detached cells and merozoites produced by *Pb apiG3PAT (−)* and wild type parasites at 66 and 72 h post‐infection. *Pb apiG3PAT (−)* parasites were not observed to produce detached cells or merosomes at either time point, indicating disruption of the enzyme severely compromized completion of liver stage development *in vitro*.

The reduction in *Pb apiG3PAT (−)* sporozoite infectivity hinted that as for FASII (Yu *et al.*, 2008; Vaughan *et al.*, 2009), the enzyme may be required for parasite development in the late liver stage. To investigate this, we compared the ability of *Pb apiG3PAT (−)* and wild type sporozoites to invade and develop within HepG2 hepatocytes *in vitro*. *Pb apiG3PAT (−)* and wild type sporozoites did not differ in their ability to invade hepatocytes, indicating the enzyme was not required for the initial establishment of liver stage infections (data not shown). Focusing on *Pb apiG3PAT (−)* clone 1, we then investigated whether the enzyme was required for the growth or development of liver stage parasites. First, to investigate if disruption of *Pb* apiG3PAT affected liver stage parasite growth, we performed immunofluorescence microscopy of *Pb apiG3PAT (−)* clone 1 and wild type at various times post‐infection using antibodies against the cytosolic marker HSP70 and measured parasite cross‐sectional area as described (Schrader *et al.*, 2013) in three independent experiments. No difference in mean parasite size was observed between *Pb apiG3PAT (−)* and wild type parasites at 24 h post‐infection, demonstrating the enzyme was not required for parasite growth in the early liver stage (Fig. 6A). However, at 48 and 66 h post‐infection, *Pb apiG3PAT (−)* parasites were significantly smaller than wild type as determined by a two‐tailed *t*‐test, with mean areas 22% and 26% less than the control, respectively (Fig. 6B and C, Supplementary Fig. 7). Disruption of *Pb* apiG3PAT therefore negatively impacted the growth of parasites in the late liver stage, consistent with the requirement for both *Py* apiG3PAT (Lindner *et al.*, 2014) and FASII for normal growth at this stage in rodent malaria models (Yu *et al.*, 2008; Vaughan *et al.*, 2009).

Next, to investigate whether disruption of *Pb* apiG3PAT affected the ability of late liver stage parasites to complete development, we assessed their capacity to produce detached cells and merosomes, which are the final liver stage forms normally observed *in vitro* (Sturm *et al.*, 2006). To examine the formation of detached cells and merosomes, we collected culture supernatants from hepatocytes at 66 and 72 h post‐infection, stained briefly with Hoechst nuclear stain, then counted these exoerythrocytic forms by live fluorescence microscopy on three separate occasions. Consistent with previous reports (Nagel *et al.*, 2013), wild type parasites produced the majority of detached cells and merosomes at 66 h post‐infection, with smaller numbers of these forms detected at the 72 h (Fig. 6D). By contrast, *Pb apiG3PAT (−)* clone 1 was never seen to produce detached cells or merosomes at either 66 or 72 h. Disruption of *Pb* apiG3PAT therefore severely compromised the ability of parasites to complete liver stage development *in vitro*. This in turn suggested the reduced infectivity of *Pb apiG3PAT (−)* sporozoites resulted from defects in liver stage merozoite production, although evidently a small number of merozoites were still produced *in vivo*, consistent with reports for the *P. berghei* FASII null mutants (Yu *et al.*, 2008; Nagel *et al.*, 2013).

### Pb *apiG3PAT is required for normal apicoplast and nuclear development in the late liver stage, but is not critical for expression of merozoite surface protein 1*

**Figure 7:**
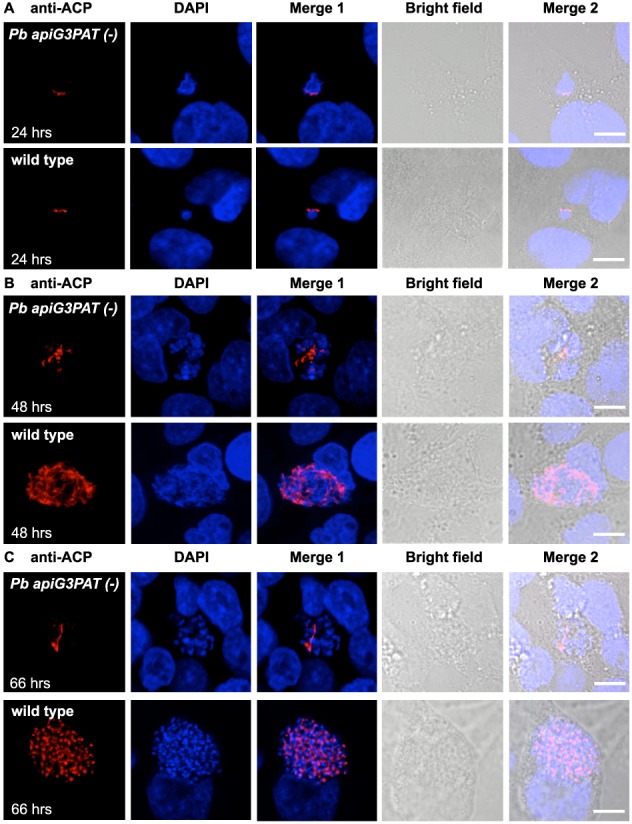
*Pb apiG3PAT (−)* parasites have impaired apicoplast and nuclear development in the late liver stage. A. Immunofluorescence microscopy of *Pb apiG3PAT (−)* and wild type liver stage parasites using antibodies against the apicoplast marker ACP and DAPI nuclear stain at 24 h post‐infection. *Pb apiG3PAT (−)* and wild type parasites show similar patterns of ACP and DAPI staining at this time point, indicating apicoplast and nuclear development are not affected by disruption of the enzyme in the early liver stage. B and C. Immunofluorescence microscopy of *Pb apiG3PAT (−)* and wild type liver stage parasites using anti‐ACP antibodies and DAPI nuclear stain at 48 and 66 h post‐infection. *Pb apiG3PAT (−)* parasites have noticeably smaller apicoplasts and fewer nuclei than wild type at both time points, indicating apicoplast and nuclear development are severely impaired by disruption of the enzyme in the late liver stage. Scale bar 10 µm.

Having established that *Pb apiG3PAT (−)* parasites showed similar defects in late liver stage growth and maturation to FASII null mutants in rodent malaria models (Yu *et al.*, 2008; Vaughan *et al.*, 2009), we next tested whether disruption of the enzyme reproduced any of the other defects reported for these mutants (Pei *et al.*, 2010; Annoura *et al.*, 2012; Nagel *et al.*, 2013). To explore whether disruption of *Pb* apiG3PAT similarly affected apicoplast and nuclear development in the late liver stage, we performed immunofluorescence microscopy of *Pb apiG3PAT (−)* clone 1 and wild type parasites with antibodies against ACP and DAPI nuclear stain at various time points post‐infection. No difference was observed between *Pb apiG3PAT (−*) and wild type parasites at 24 h post‐infection, demonstrating the enzyme was not required for apicoplast or nuclear development in the early liver stage (Fig. 7A). By contrast, *Pb apiG3PAT (−)* parasites had noticeably smaller apicoplasts and fewer nuclei than wild type at 48 and 66 h (Fig. 7B and C). Moreover, whereas wild type parasites had typically undergone schizogony and the apicoplast and nuclei had divided into daughter merozoites by 66 h, *Pb apiG3PAT (−)* parasites were never seen to progress to this stage. This further supported our finding that disruption of *Pb* apiG3PAT severely impacted liver stage merozoite production, and suggested that like FASII (Yu *et al.*, 2008; Vaughan *et al.*, 2009; Pei *et al.*, 2010), the enzyme may be directly or indirectly required for apicoplast and nuclear development in the late liver stage.

**Figure 8:**
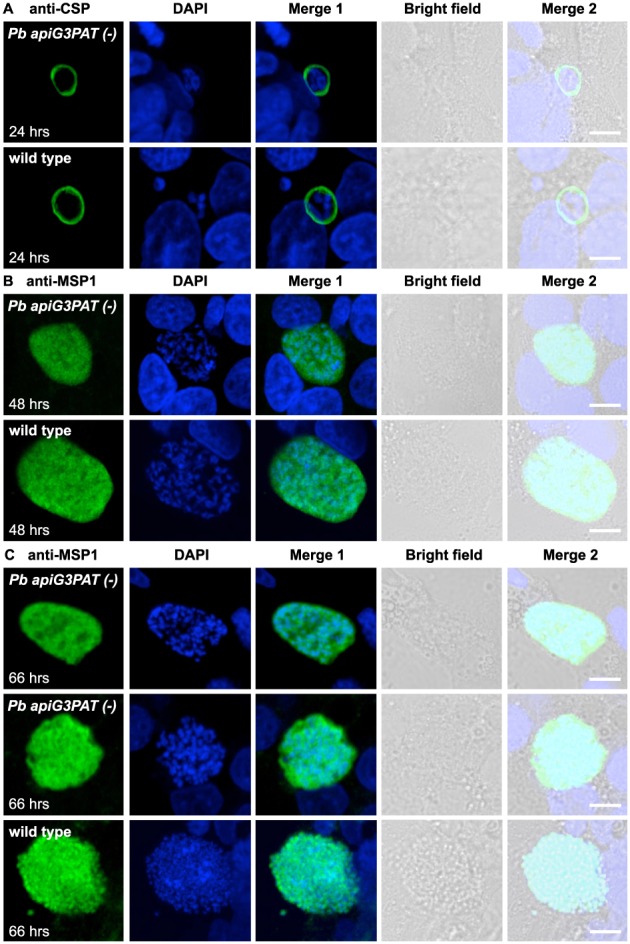
*Pb apiG3PAT (−)* parasites show altered plasma membrane morphology in the late liver stage but still express merozoite surface protein 1. A. Immunofluorescence microscopy of *Pb apiG3PAT (−)* and wild type parasites using antibodies against the CSP at 24 h post‐infection. *Pb apiG3PAT (−)* and wild type parasites show similar patterns of CSP staining at this time point, indicating plasma membrane morphology is not affected by disruption of the enzyme in the early liver stage. B. Immunofluorescence microscopy of *Pb apiG3PAT (−)* and wild type parasites using antibodies against merozoite surface protein 1 (MSP1) at 48 h post‐infection. Similar patterns of MSP1 expression are observed in *Pb apiG3PAT (−)* and wild type parasites, indicating disruption of the enzyme does not markedly affect plasma membrane morphology or MSP1 expression at this stage. C. Immunofluorescence microscopy of *Pb apiG3PAT (−)* and wild type parasites using anti‐MSP1 antibodies at 66 h post‐infection. *Pb apiG3PAT (−)* and wild type parasites differ noticeably in their pattern of MSP1 staining at this time point, suggesting disruption of the enzyme affects formation of merozoite membranes in the late liver stage. Importantly, although there was only one instance where evidence of plasma membrane invagination was observed for *Pb apiG3PAT (−)* parasites (middle panel), MSP1 expression was still readily detected in the line. DNA with DAPI. Scale 10 µm.

Next, we investigated whether as in FASII null mutants in rodent models (Yu *et al.*, 2008; Vaughan *et al.*, 2009; Pei *et al.*, 2010; Annoura *et al.*, 2012; Nagel *et al.*, 2013), disruption of *Pb* apiG3PAT affected the expression of MSP1 or its localization to the plasma membrane in late liver stage parasites. For this, we used immunofluorescence microscopy to localize MSP1 in *Pb apiG3PAT (−)* clone 1 and wild type parasites at 48 and 66 h post‐infection. To enable the plasma membrane of early liver stage parasites to be examined, we also performed immunofluorescence microscopy of parasites at 24 h post‐infection using antibodies against CSP as previously described (Lindner *et al.*, 2014). No difference was observed between *Pb apiG3PAT (−)* and wild type in CSP staining at 24 h, confirming disruption of the enzyme did not impact parasite growth or plasma membrane morphology in the early liver stage (Fig. 8A). Surprisingly, we also failed to detect any perceivable difference in MSP1 staining at 48 h, with *Pb apiG3PAT (−)* parasites again appearing smaller than wild type, but nonetheless showing a diffuse pattern of MSP1 staining similar to the control (Fig. 8B). This indicated that although late liver stage growth was reduced in *Pb apiG3PAT (−)* parasites, the expression and localization of MSP1 were not noticeably altered, contrasting with the phenotype reported for FASII null mutants (Yu *et al.*, 2008; Vaughan *et al.*, 2009; Pei *et al.*, 2010; Annoura *et al.*, 2012; Nagel *et al.*, 2013). Consistent with our finding that loss of *Pb apiG3PAT* severely impaired merozoite production, we did observe differences between the lines at 66 h, with MSP1 staining typically detected around individual merozoites in the wild type, but remaining patchy or indicative of only limited plasma membrane invagination in *Pb apiG3PAT (−)* parasites (Fig. 8C). This suggested that as for FASII in the rodent malaria models, *Pb* apiG3PAT was likely involved in synthesizing lipids for the developing merozoite membranes. However, as MSP1 staining was still readily detected in *Pb apiG3PAT (−*) parasites, it again indicated that MSP1 expression was not impacted to the same extent as for the *P. berghei* FASII null mutants (Yu *et al.*, 2008; Annoura *et al.*, 2012).

**Figure 9:**
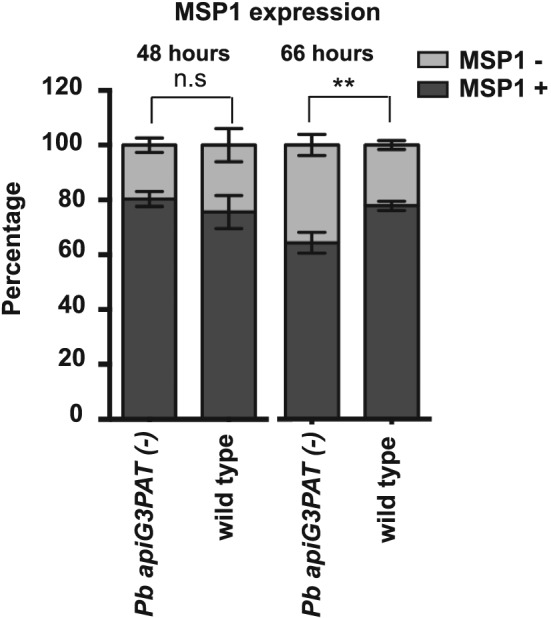
*Pb apiG3PAT (−)* parasites show only a minor defect in merozoite surface protein 1 expression in the late liver stage. Proportion of *Pb apiG3PAT (−)* and wild type parasites expressing merozoite surface protein 1 (MSP1) at 48 and 66 h post‐infection. *Pb apiG3PAT (−)* and wild type parasites do not differ significantly in the proportion of MSP1 positive parasites at 48 h post infection as determined by a two tailed *t*‐test (n.s., non‐significant). The proportion of MSP1 positive parasites in the *Pb apiG3PAT (−)* and wild type lines does differ significantly at 66 h post‐infection (**, *p*‐value <0.05), but as still over 60% of *Pb apiG3PAT (−)* parasites express MSP1 at this time point, this defect appears to be relatively minor. Error bars show mean of three biological replicates ± standard error.

To more quantitatively compare the proportions of MSP1‐expressing parasites in the *Pb apiG3PAT (−)* clone 1 and wild type lines, we performed immunofluorescence microscopy of late liver stage parasites using antibodies against both MSP1 and the parasitophorous vacuole marker UIS4 (Mueller *et al.*, 2005). Using UIS4 staining to identify parasites, we scored the proportion that was MSP1‐positive at 48 and 66 h in three independent experiments. Consistent with our observation using MSP1 antibodies alone, no significant difference was observed between *Pb apiG3PAT (−)* and wild type at 48 h, with over 75% of parasites scored as MSP1 positive in each line (Fig. 9, Supplementary Fig. 8). A modest but significant decrease was observed between *Pb apiG3PAT (−)* and wild type at 66 h, with the mean proportion of MSP1 positive parasites found to be 64% and 78%, respectively (Fig. 9, Supplementary Fig. 8). However, as this equated to only an 18% reduction relative to wild type, the MSP1 phenotype observed for *Pb apiG3PAT (−)* parasites was still far more mild than reported for the *P. berghei* FASII null mutants (Yu *et al.*, 2008; Annoura *et al.*, 2012). This suggested that *Pb* apiG3PAT and FASII likely differed in their contribution to the synthesis of the MSP1 GPI anchor, and that the requirement for the enzyme for late liver stage development instead likely reflects its role in the synthesis of other essential lipid species. In this respect it is important to note that the *P. falciparum* MSP1 GPI anchor typically contains two palmitate (C16:0) fatty acids in its diacylglycerol moiety, and an additional myristate (C14:0) fatty acid linked directly to the inositol ring (Gerold *et al.*, 1996). Assuming the *P. berghei* MSP1 GPI anchor is composed of the same fatty acids, the greater reliance on FASII would be consistent with the pathway contributing the myristate (C14:0) and potentially also fatty acids for its diacylglycerol moiety, whereas *Pb* apiG3PAT may only be required for production of the latter or not necessary for synthesis of the anchor at all. Our findings are therefore consistent with FASII fatty acids taking multiple pathways out of the apicoplast into lipids, with some fatty acids reliant on *Pb* apiG3PAT for incorporation into precursors for membrane lipid synthesis, while others are putatively exported prior to being incorporated into lipids such as the MSP1 GPI anchor.

## Conclusions

In this study, we have characterized the *P. falciparum* and *P. berghei* apicoplast G3PAT and assessed the phenotype of deletion mutants to investigate how the enzyme contributes to linking FASII with membrane lipid production in each *Plasmodium* species. We confirm apicoplast targeting of the *P. falciparum* and *P. berghei* enzyme and demonstrate the activity of *Pf* apiG3PAT by complementation, providing evidence that FASII fatty acids can contribute to the initial step in phosphatidic acid synthesis in the apicoplast of both human and rodent malaria parasites. We find that disruption of the enzyme largely mirrors the phenotype of the FASII null mutants in each species, with no apparent defect observed for the *Pf* apiG3PAT mutant in blood stage parasites, but severe defects observed for the *Pb* apiG3PAT mutant in growth and merozoite formation in the late liver stage that ultimately result in markedly decreased parasite infectivity. We also find indirect evidence for FASII fatty acid utilization via other pathways in both species, as suggested by the blood stage growth of *Pf* apiG3PAT mutant in lipid‐depleted media conditions and the relatively mild MSP1 phenotype observed for the *Pb* apiG3PAT mutant in the late liver stage.

Together, these findings extend upon previous research on the *P. yoelii* apicoplast G3PAT, and reveal new information about how pathways in both the apicoplast and ER contribute to incorporating FASII fatty acids into precursors for membrane lipids such as the MSP1 GPI anchor. We provide important confirmation that the requirement for FASII for late liver stage development in rodent malaria models likely reflects a need for fatty acids for membrane lipid production, and demonstrate that fatty acid synthesis and scavenging pathways can differ in their contribution to certain lipid species. As human and rodent malaria parasites differ in their requirement for FASII across the parasite life cycle, it is not yet clear how closely these findings will be echoed in *P. falciparum*. However, should future experiments reveal that FASII and *Pf* apiG3PAT are similarly required for the *P. falciparum* liver stage, we anticipate this information will help guide further research and assist in identifying the most strategic aspects of lipid metabolism to target for malaria prophylaxis.

## Experimental Procedures

### *Routine maintenance of* P. falciparum

*P. falciparum* D10 wild type parasites and transgenic lines were maintained as previously described (Trager and Jensen, 1976) at 2% haematocrit in RPMI‐HEPES supplemented with AlbuMAX II (Gibco) for all experiments unless otherwise stated.

### *Generation and analysis of* Pf apiG3PAT_1–70_ gfp *transgenic parasites*

The region encoding the predicted *Pf* apiG3PAT apicoplast targeting sequence was amplified from *P. falciparum* genomic DNA using primers P1 and P2 (Supplementary Table 1) and introduced into the pHBlR vector between the *Pf*HSP86 promotor and GFP coding sequence as previously described (van Dooren *et al.*, 2005). The resulting *Pf apiG3PAT_1–70_ gfp* vector was transfected into ring stage parasites using the standard electroporation protocol (Wu *et al.*, 1995; Crabb and Cowman, 1996). Parasites were then cultured in the presence of 2.5 µg ml^−1^ blasticidin S (Invitrogen) to select for episomal maintenance of the vector.

Live microscopy of *Pf apiG3PAT_1–70_ gfp* parasites was performed by staining cultures briefly with 1 µg ml^−1^ 4′,6‐diamidino‐2‐phenylindole (DAPI), before immobilizing cells in media on glass coverslips pre‐treated with 0.1% polyethyleneimine. Images were acquired using a Leica SP2 inverted confocal microscope at ambient temperature, and merged and contrast adjusted using ImageJ software (NCBI).

Immunofluorescence microscopy of *Pf apiG3PAT_1–70_ gfp* parasites was performed by fixing in paraformaldehyde/glutaraldehyde and permeabilizing in Triton X‐100 as previously described (Tonkin *et al.*, 2004) but without the sodium borohydride treatment step. Labelling was performed with mouse anti‐GFP (Roche), rabbit anti‐ACP (our laboratory (Waller *et al.*, 2000)), Alexa Fluor goat anti‐mouse‐488 and Alexa Fluor goat anti‐rabbit‐546 (Molecular Probes). Samples were then stained with 1 µg ml^−1^ DAPI and mounted in Fluorescence Mounting Medium (DAKO) before imaging as above.

### *Bacterial complementation assay and mutagenesis of* Pf *apiG3PAT*

The *Pf* apiG3PAT coding sequence (without apicoplast targeting sequence) was amplified from *P. falciparum* genomic DNA using primers P3 and P4 (Supplementary Table 1) and introduced into the pQE‐30 Xa vector (Qiagen) immediately downstream of the hexahistidine tag. For the positive control, the coding sequence of the wild type bacterial G3PAT was amplified from *E. coli* genomic DNA using primers P5 and P6 (Supplementary Table 1) and cloned into the vector as above. Vectors were transformed into the *E. coli plsB26* mutant alongside the pREP4 co‐vector (Qiagen), and clones were selected and grown under permissive conditions to allow all isolates to be propagated. To assess for complementation, equivalent cell numbers were transferred to non‐permissive M56LP media without glycerol (Bell, 1974), then growth at 37 C was assessed by monitoring optical density at 600 nm. All assays were performed in duplicate on different days using independently isolated colonies, with two technical replicates for each time point.

Western blot analysis of transformed bacteria was performed by growing isolated clones under permissive conditions, pelleting cells by centrifugation, then lyzing by freeze/thawing and boiling in SDS loading buffer. Proteins were separated by SDS‐PAGE and transferred to nitrocellulose, then membranes were probed using mouse anti‐hexahistidine (Thermo Scientific) and goat anti‐mouse‐horseradish peroxidase (Thermo Scientific). Protein bands were detected using ECL Western Blotting Substrate (Pierce) and images were acquired using a BioRad ChemiDoc Imager.

Site directed mutagenesis of the *Pf* apiG3PAT ‘HX_4_D’ motif was performed by Mutagenix Inc., and activity of the mutated versions of the protein assessed by repeating the bacterial transformation, complementation assay and Western blot analysis as above.

### *Generation and analysis of* Pf *apiG3PAT (−) parasites*

Two regions of the *Pf* apiG3PAT coding sequence were amplified from *P. falciparum* genomic DNA using primers P7, P8, P9 and P10 (Supplementary Table 1) and introduced into the pCC‐1 transfection vector (Maier *et al.*, 2008). The resulting vector was transfected into ring stage parasites using the standard electroporation protocol (Wu *et al.*, 1995; Crabb and Cowman, 1996), then selection was performed with 5 nM WR99210 (Jacobus Pharmaceuticals) and 1 μM 5‐fluorocytosine (Sigma) as previously described (Maier *et al.*, 2006). Disruption of the *Pf* apiG3PAT locus in the two independently derived parasite populations was confirmed by PCR using primers P11, P12 and P13, with primers T1 and T2 used as controls (Supplementary Table 1).

Modification of the *Pf* apiG3PAT locus in the principal line examined in this study was additionally confirmed by whole genome sequencing as previously described (Straimer *et al.*, 2012). Briefly, PCR‐free libraries were prepared using NEBNext DNA Library reagents (NEB) and NEXTflex DNA Barcodes (Bioo Scientific). Eight libraries were multiplexed with 8% PhiX control and run across two lanes on the Illumina HiSeq 2500 system using single‐end sequencing. Sequencing data was analysed using Galaxy (Giardine *et al.*, 2005; Blankenberg *et al.*, 2010; Goecks *et al.*, 2010). Reads were mapped to the *P. falciparum* 3D7v.10.0 reference genome (http://plasmodb.org/common/downloads/release‐10.0/Pfalciparum3D7/) using the Burrows‐Wheeler Alignment tool (Li and Durbin, 2009). Variants were called using Freebayes (Garrison and Marth, 2012), filtered for quality and read depth with GATK tools (Auwera *et al.*, 2013) (Quality > 500, Read Depth >75), and annotated using SnpEff (Cingolani *et al.*, 2012) (PF3D7v9.1 genome).

Immunofluorescence microscopy of parasites was performed using anti‐ACP, Alexa Fluor goat anti‐rabbit secondary antibodies and DAPI as above. Transmission electron microscopy was performed with trophozoite stage parasites previously described (Sturm *et al.*, 2015). Briefly, infected RBCs were isolated using MACS magnetic separation columns (Miltenyi Biotech) and fixed in 2.5% glutaraldehyde in cacodylate buffer, then treated with 2% osmium tetroxide and 1.5% potassium ferocyanide followed by 1% thyocarbohydrazide. Samples were further stained with 2% uranyl acetate and Walton's lead aspartate before embedding in Procure 812 resin. Sections of approximately 70–100 nm were then cut and imaged using a Philips CM120 BioTWIN Transmission Electron Microscope and Gatan Multiscan Model 971 digital camera.

Blood stage growth assays in standard media were performed by monitoring the replication of synchronous parasites over two asexual cycles as previously described (Mitamura *et al.*, 2000; Mi‐Ichi *et al.*, 2006). Prior to the assay, parasites were synchronized by two treatments with 5% sorbitol, and then allowed to mature into trophozoites before being transferred to assay dishes. Media was replaced daily, and parasitemia monitored by thin Giemsa‐stained blood smears. Growth assays in lipid‐depleted media were performed by synchronizing parasites as above, then washing parasites before transferring to lipid‐depleted media to initiate the assay. Lipid‐depleted media was prepared as previously reported (Botté *et al.*, 2013) by replacing the lipid‐rich AlbuMAX II component of standard media with an equivalent amount of fatty acid free bovine serum albumin (Sigma) and 30 μM palmitic acid (C16:0; Sigma) and 45 μM oleic acid (C18:1; Sigma). All assays were performed in triplicate on different days, and with a minimum of 400 RBCs counted per line time point.

Fatty acid profiling of magnetically isolated infected RBCs was performed as previously described (MacRae *et al.*, 2014) with minor modifications. Briefly, cultures were pelleted by centrifugation and metabolism was quenched by immersion in dry ice/methanol slurry. Infected red blood cells were isolated using MACS magnetic separation columns (Miltenyi Biotech) at 4 C, before lipids were extracted from 1.0 to 2.0 × 10^6^ cells using chloroform:methanol (2:1 v/v, spiked with 25 nmol lauric acid as an internal standard) by periodic sonication for 1 h in a refrigerated cold room. Samples were then centrifuged, and supernatants transferred to new tubes and dried under nitrogen. Pellets were re‐extracted using methanol:water (2:1 v/v), and the second supernatants were then combined with the first extract and dried then stored at −80 C. Prior to GC‐MS, extracts were partitioned into chloroform:methanol:water (1:3:3 v/v) and the apolar (lipid) phase was dried, derivatized to fatty acid methyl esters and analysed on an Agilent 7890B‐5977A GC‐MS system using a DB‐5MS‐DG column as previously described (MacRae *et al.*, 2012). Data analysis was performed using MassHunter (Agilent) and fatty acid species were identified and quantified by comparison to authentic standards. Experiments were repeated with four biological replicates, and statistical significance between mean fatty acid abundances evaluated by *t*‐test with correction for multiple testing using Prism v 6.02 (GraphPad).

### *Experimental animals and routine maintenance of* P. berghei

Swiss Webster mice of four to six weeks of age were used for all experiments, and were sourced from the University of Melbourne Zoology Animal Facility or Monash University Animal Research Platform. All experiments were conducted in accordance with the local Prevention of Cruelty to Animals legislation and the University of Melbourne Animal Ethics Committee guidelines under ethics permits 1112043.1 and 1413078.

Mice were infected with *P. berghei* ANKA wild type or transgenic parasite lines by intraperitoneal injection, and parasitemia was monitored by thin Giemsa‐stained smears. Numbers of male gametocytes were assessed by monitoring exflagellation as described (Sturm *et al.*, 2015), and mice were deemed suitable for mosquito infection when >3 exflagellation events per 1000 RBCs were observed. Adult female *Anopheles stephensi* mosquitoes aged 3 to 7 days were infected by feeding on parasitized mice until engorged. Mosquitoes were maintained on 10% sucrose at 27 C and 80% humidity, and naive mice were infected after 21 days by biting or intravenous injection of sporozoites into the tail vein.

### *Infection of HepG2 cells with* P. berghei *sporozoites*

HepG2 cells were grown at 37 C and 5% carbon dioxide in Advanced MEM medium (Gibco) containing 10% heat‐inactivated fetal bovine serum, 2 mM GlutaMAX (Gibco), 1% penicillin/streptomycin (Hyclone) and a variable concentration of amphotericin B (Hyclone). Cells were seeded onto glass coverslips in 24‐well plates or glass‐bottomed culture dishes pre‐treated with rat tail collagen type I (Sigma), and allowed to grow overnight before infection with sporozoites. Media was then changed 1–2 h after infection and twice daily thereafter for all experiments.

### *Generation and analysis of* Pb *apiG3PAT ha gfp parasites*

The *Pb apiG3PAT* coding sequence (minus stop codon) was amplified from *P. berghei* genomic DNA using primers P14 and P15 and introduced into the pREP3 transfection vector, which was derived from the pL0006 vector (Malaria Research and Reference Reagent Resource Center) by addition of the GFP coding sequence downstream of the multiple cloning site. The resulting vector was linearized by cutting with BstEII, then transfected into schizont stage parasites using the Nucleofector device (Lonza) as previously described (Janse *et al.*, 2006). Pyrimethamine‐resistant parasites were recovered and clonal lines obtained by limiting dilution in 10 mice. The correct integration of the vector into the *Pb* apiG3PAT locus was confirmed by PCR using primers P16, P17 and P18, with primers T1 and T2 used as controls (Supplementary Table 1).

Live imaging of liver stage parasites was performed 48 h post‐infection. Cells were stained briefly with 5 µg ml^−1^ Hoechst 33342 (Life Technologies), then imaged using a Leica SP5 inverted laser scanning confocal microscope in a 37 C temperature controlled chamber. Images were taken sequentially for the two channels and processed using ImageJ software (NCBI).

Immunofluorescence microscopy of liver stage parasites was performed 48 post‐infection. Cells were washed in PBS, fixed in 4% paraformaldehyde, washed again and permeabilized in 0.15% Triton X‐100 before blocking in 3% bovine serum albumin. Labelling was performed with mouse anti‐GFP (Roche), rabbit anti‐FabI (gift from David Fidock; used as anti‐ACP antibodies were temporarily unavailable), Alexa Fluor goat anti‐mouse‐488 and anti‐rabbit‐546 secondary antibodies (Molecular Probes). Samples were then stained with 1 µg ml^−1^ DAPI, mounted in Fluorescence Mounting Medium (DAKO), and imaged using a Leica SP5 at ambient temperature as above.

### *Generation and analysis of* Pb *apiG3PAT (−) parasites*

Regions of the *Pb* apiG3PAT coding sequence and 3′ UTR were amplified from *P. berghei* genomic DNA using primers P19, P20, P21 and P22 (Supplementary Table 1) and introduced into the pL0006 vector (Malaria Research and Reference Reagent Resource Center). The vector was linearized with SacII and ApaI, then transfected into parasites and clonal lines obtained by limiting dilution as above. The correct integration of the vector into the *Pb apiG3PAT* locus was confirmed by PCR using primers P16, P17 and P23, with primers T1 and T2 used as controls (Supplementary Table 1).

Blood stage parasite growth assays were performed as previously described (Sturm *et al.*, 2015) by intravenously injecting three sets of two mice with 1.0 × 10^5^ infected RBCs, then monitoring parasitemia daily by thin Giemsa‐stained smears.

Male gametocyte and mosquito stage development of parasites were assessed by determining the mean exflagellation rate and mean number of ookinetes, oocysts and salivary gland sporozoites for five separate infections. Exflagellation rate was determined as previously described (Sturm *et al.*, 2015), then mosquito cages were infected and 10 individuals sacrificed at each time point. For ookinetes, blood boluses were isolated from mosquitoes 20–22 h post‐infection, pooled and stained with mouse anti‐p28 (gift from Robert Sinden) and goat anti‐mouse Alexa Fluor 488 (Molecular Probes), then parasites were counted as described (Sturm *et al.*, 2015) on a haemocytometer using an Olympus IX73 epifluorescence microscope. For oocysts, midguts were isolated from mosquitoes 14–15 days post‐infection, stained with 2% Mercurochrome (Sigma), then viewed using an Olympus BH‐2 light microscope. For sporozoites, salivary glands were isolated 21–22 days post‐infection, disrupted in PBS, and then parasites were counted on a haemocytometer using an Olympus CK2 microscope.

Sporozoite infectivity *in vivo* was assessed by measuring the time to patency following intravenous injection of 1000 or 10 000 sporozoites or exposure to 10 infected mosquitoes for ten minutes. Parasitemia was monitored on days 3–14 post‐infection by thin Giemsa‐stained smears as described (Nagel *et al.*, 2013). Smears were viewed using an Olympus BH‐2 light microscope with 40× oil objective, and patency was judged by scanning a minimum of 20 adjacent fields of view as previously reported (Lindner *et al.*, 2014).

Liver stage parasite size was measured by performing immunofluorescence microscopy of parasites at 24, 48 and 66 h post‐infection. Cells were fixed as above and labelled with mouse anti‐HSP70 (gift from Moriya Tsuji), goat anti‐mouse Alexa Fluor 488 (Molecular Probes) and DAPI, before mounting in Fluorescence Mounting Medium (DAKO). Imaging was performed using an OMX V4 Blaze in wide field deconvolution mode, with z‐slices acquired across the entire depth of the parasites. Image deconvolution and maximum projection were performed using softWORx software (Applied Precision), and parasite cross‐sectional areas calculated using ImageJ (NCBI). Experiments were repeated three times with >50 parasites imaged per replicate, and statistical analysis was performed using Prism version 6.0 (GraphPad).

Detached cell and merosome formation by parasites was assessed by collecting culture supernatants 66 and 72 h post‐infection. Cells were stained briefly with 5 µg ml^−1^ Hoechst 33342 (Life Technologies), the counted using the Leica SP2 inverted microscope in epifluorescence mode. Experiments were repeated three times, with a minimum of four wells counted on each occasion.

Immunofluorescence microscopy of liver stage parasites to examine apicoplast and plasma membrane morphology was performed by fixing and labelling with rabbit anti‐ACP, mouse anti‐CSP (gift from Louis Schofield) or mouse anti‐MSP1 (gift from Paul Gilson), then with Alexa Fluor secondary antibodies and DAPI before mounting as above. Images were acquired with a Leica SP2 inverted confocal microscope and processed using ImageJ (NCBI).

Immunofluorescent microscopy of liver stage parasites to quantify MSP1 staining was performed by fixing and staining with mouse anti‐UIS4 (gift from Photini Sinnis) and anti‐MSP1 antibodies, then with secondary antibodies and DAPI before mounting and imaging as above. Experiments were repeated three times with >100 parasites counted per replicate, and statistical analysis was performed using Prism version 6.0 (GraphPad).

## Supporting information


**Table S1**. Primers used in this study.
**Table S2**. Mean relative fatty acid abundance in total lipid extracts from infected RBCs isolated from cultures of *Pf apiG3PAT (−)* or wild type parasites.
**Fig. S1**. Alignment of *Pf* apiG3PAT and *Pb* apiG3PAT showing the predicted apicoplast targeting sequence and ‘HX4D’ motif characteristic of glycerol 3‐phosphate acyltransferases.
**Fig. S2**. Generation of *Pf apiG3PAT (−)* parasites.
**Fig. S3**. Disruption of *Pf* apiG3PAT does not affect blood stage cell cycle progression on standard or lipid‐depleted media.
**Fig. S4**. *Pb* apiG3PAT tagging strategy and confirmation by PCR.
**Fig. S5**. Live fluorescence microscopy of *Pb apiG3PAT ha gfp* liver stage parasites.
**Fig. S6**. *Pb* apiG3PAT knockout strategy and confirmation by PCR.
**Fig. S7**. Representative immunofluorescence images of *Pb apiG3PAT (−)* and wild type parasites as used for measuring liver stage cell size.
**Fig. S8**. Representative immunofluorescence images of *Pb* apiG3PAT *(−)* and wild type parasites as stained for scoring expression of merozoite surface protein 1 (MSP1) in the late liver stage.

Supporting info item
