# Supplementary material for: Characterization of the Plasmodium falciparum and P. berghei glycerol 3‐phosphate acyltransferase involved in FASII fatty acid utilization in the malaria parasite apicoplast
Source: Cell Microbiol. 2016 Aug 1;19(1):e12633. doi: 10.1111/cmi.12633 (PMC5213128; doi:10.1111/cmi.12633)
Supplement: Supplementary file 1 — Supporting info item [file CMI-19-0-s001.pdf]

Supplementary Table 1: Primers used in this study

Primer	Sequence
P1	5'-CACCATGATTAAATTTTCTGTTCTTTCC-3'
P2	5'-AAATGTTATAATTTTACTTTTTTTACATG-3'
P3	5'-AAAAGCTTTTTCCTAAATATATAGATAAAAG-3'*
P4	5'-AAAAGCTTACTGATTTGTTTATACAATTG-3'
P5	5'-AAAAGCTTTCCGGCTGGC-CACGAATTTACTAC-3'
P6	5'-AAAAGCTTCCCTTCGCCCTGCGTCGCAC-3'
P7	5'-AACCATGGATGATTAAATTTTCTGTTCTT-3'
P8	5'-AAGAATTC-ATGGTGATTACTAAATATTAT-3'
P9	5'-AACTTAAGTGAAGCAGATGCAAATAT-3'
P10	5'-AACCGCGGTCAACTGATTTGTTTATACA-3'
P11	5'-TATAATTGTCACAGTATGATTAAATTTTCTGTTC-3'
P12	5'-GCATCTGCTTCAATATGGTGATTAC-3'
P13	5'-CATATTTATTAAATCTAGAATTCATGGTGATTAC-3'
P14	5'-AAAGATCTATGGGCTATTTGCATAAATC-AGTATC-3'
P15	5'-AACCTAGG*AGCGTAATCTGGAACATCGTATGGGTA*TATCTTCTTATATAGATCATTAACATAGTC-3'#
P16	5'-CATGCATGTGTTAGCTTATTTTAG-3'
P17	5'-CCAGTTGTAAGGATTATTCACTC-3'
P18	5'-CTTCAGCACGTGTCTTGTAG-3'
P19	5'-AAGAATTCATGGGCTATTTGCATAAATCAGTATC-3'
P20	5'-AAGCGGCCGCGATGATTACTAAATATAAATACATTGTGTC-3'
P21	5'-AACCGCGGCCGCCTCATTTAAAAGAAGAAAA-3'
P22	5'-AAGGGCCCTGTCATAGTCCATTTCCCTTCA-3'
P23	5'-CCGCTCAGGAACGAATTTAG-3'
T1	5'-TGGAGCAGGAAATAACTGGG-3'
T2	5'-ACCTGACATAGCGGCTGAAA-3'

*Restriction sites underlined

#HA tag in italics

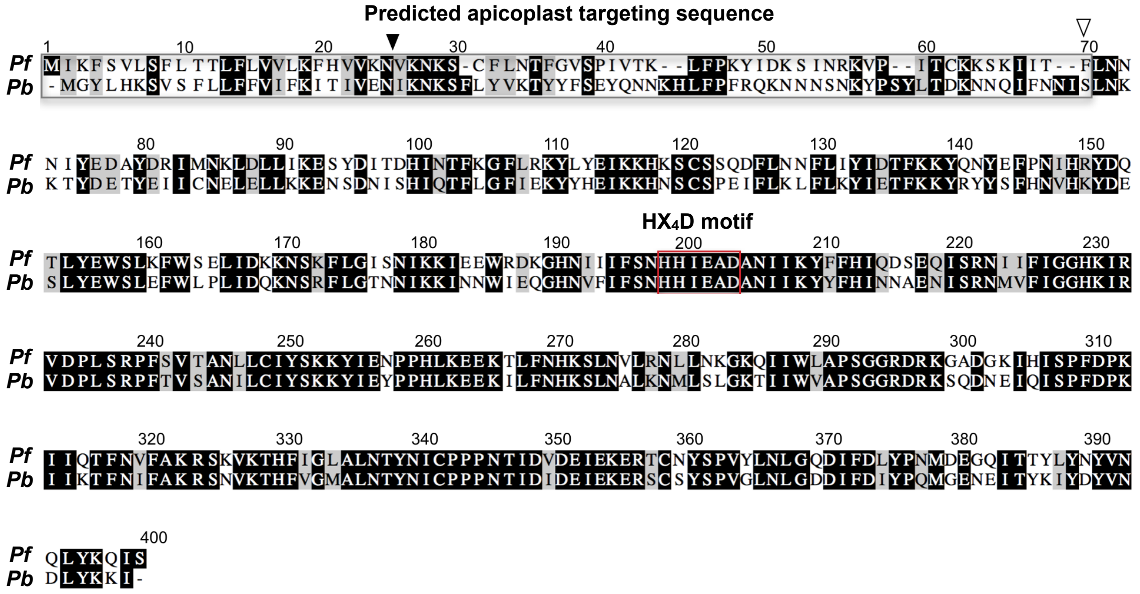

Supplementary Figure 1. Alignment of *Pf* apiG3PAT and *Pb* apiG3PAT showing the predicted apicoplast targeting sequence and 'HX$_4$D' motif characteristic of glycerol 3-phosphate acyltransferases. The predicted *Pf* apiG3PAT apicoplast targeting sequence (grey box) was deduced from the outputs of the SignalP, PATS and PlasmoAP targeting prediction algorithms and manual inspection of the protein sequence. The signal peptide component of the *Pf* apiG3PAT targeting sequence was predicted to span residues 1-25 based on the putative cleavage point identified by SignalP (closed arrowhead). The transit peptide component of the targeting sequence was predicted to span residues 26-70 based on analysis of amino acid composition and comparison to known transit peptides. The predicted transit peptide cleavage point (closed arrowhead) indicates the proposed boundary between the apicoplast targeting sequence and start of the mature protein. The predicted *Pb* apiG3PAT targeting sequence was deduced from the output of SignalP and comparison to the *Pf* apiG3PAT sequence. Both *Pf* apiG3PAT and *Pb* apiG3PAT contain the conserved 'HX$_4$D' motif (red box) characteristic of the glycerol 3-phosphate acyltransferase family.

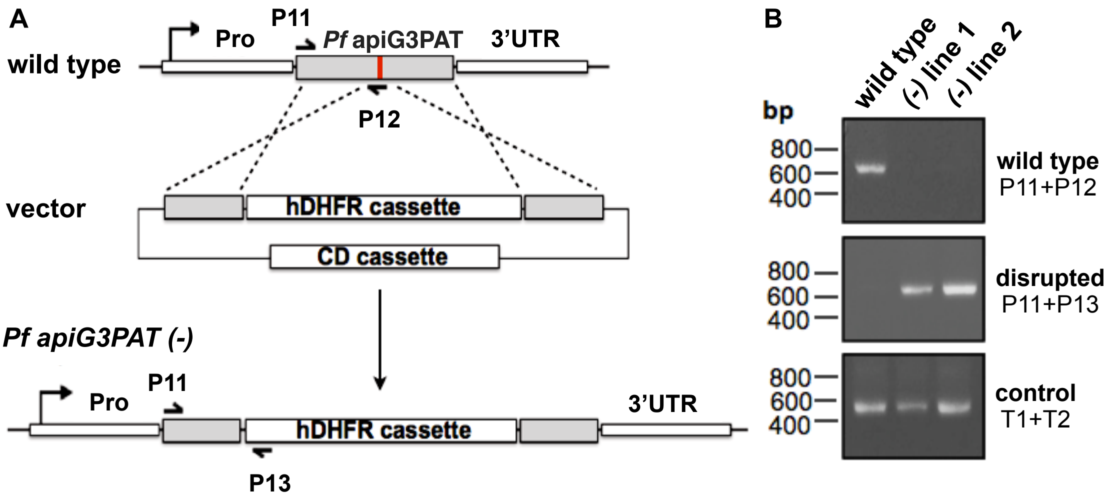

Supplementary Figure 2. Generation of *Pf apiG3PAT (-)* parasites. A. Diagram of the strategy used to generate *Pf apiG3PAT (-)* parasites. Following double crossover homologous recombination, the *Pf* apiG3PAT coding sequence is disrupted by the human dihydrofolate reductase (hDHFR) cassette, truncating the enzyme and eliminating the conserved 'HX_4D' motif (red). The cytosine deaminase (CD) cassette in the vector backbone enables negative selection against parasites with episomes to favor integration of the hDHRF cassette. Positions of primers used to confirm the gene disruption by PCR are shown, with primers P11+P12 expected to give a 629 bp product and primers P11+P13 a 634 bp product. B. PCR verifies disruption of the *Pf* apiG3PAT coding sequence in two independent lines (abbreviated *(-)* line 1 and *(-)* line 2). PCR products for the wild type parental line shown for comparison.

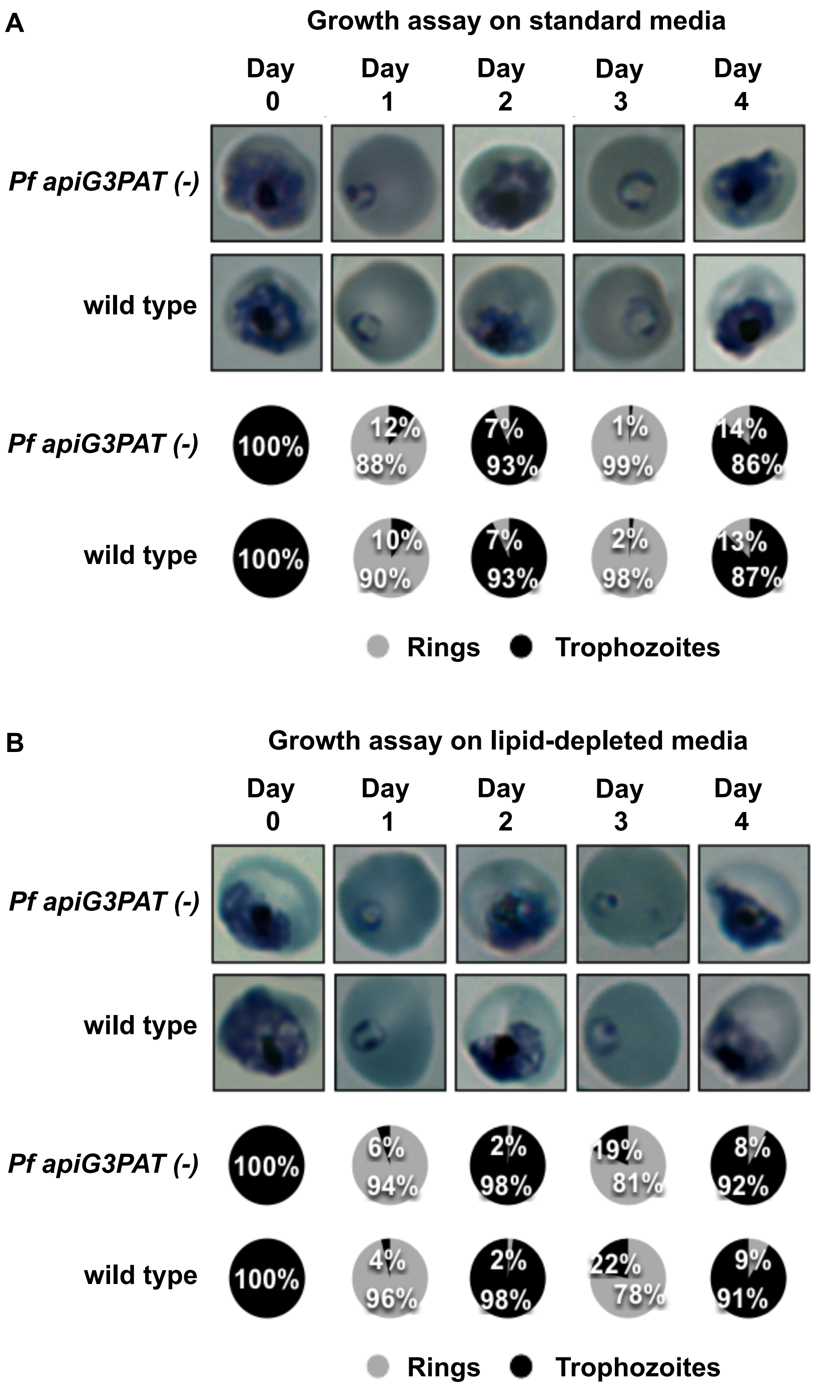

Supplementary Figure 3. Disruption of *Pf* apiG3PAT does not affect blood stage cell cycle progression on standard or lipid-depleted media. A. Representative images of Giemsa-stained *Pf apiG3PAT (-)* and wild type parasites from the growth assay on standard media. The mean percentage of ring and trophozoite stage parasites at each day of the assay are indicated in the pie charts (n=3). B. Representative images of *Pf apiG3PAT (-)* and wild type parasites from the growth assay performed on lipid-depleted media. Note schizonts were not observed in assays due to the timing of smears.

Supplementary Table 2. Mean relative fatty acid abundance in total lipid extracts from infected RBCs isolated from cultures of *Pf apiG3PAT (-)* or wild type parasites

		Pf apiG3PAT (-) (±SD[a])	wild type (±SD[a])	t-test p-value[b]
	C12:0	1.01 ±0.01	1.04 ±0.03	n.s
	C14:0	1.47 ±0.57	2.09 ±1.31	n.s
	C15:0	0.85 ±0.24	1.34 ±0.88	n.s
	C16:0	11.74 ±2.59	17.89 ±11.51	n.s
	C16:1	1.02 ±0.48	1.41 ±0.91	n.s
	C17:0	1.68 ±0.45	2.77 ±1.72	n.s
	C18:0	11.67 ±2.83	17.35 ±11.19	n.s
	C18:1a[#]	1.15 ±0.28	1.82 ±1.08	n.s
	C18:1b[#]	13.08 ±2.84	19.65 ±12.58	n.s
	C18:2a[#]	1.44 ±0.36	2.20 ±1.35	n.s
	C18:2b[#]	2.82 ±0.76	4.24 ±2.67	n.s
	C20:0	0.13 ±0.04	0.20 ±0.12	n.s
	C20:1	0.25 ±0.07	0.40 ±0.24	n.s
	C20:4	0.79 ±0.23	1.18 ±0.77	n.s
	C22:0	0.02 ±0.01	0.03 ±0.02	n.s
	C22:1	0.02 ±0.01	0.02 ±0.01	n.s
	C24:0	0.02 ±0.01	0.02 ±0.01	n.s

[a] Mean and standard deviation of four biological replicates.
[b] p-value for t-test with correction for multiple testing. All p-values greater than 0.05 were considered non-significant (n.s).
[#] 'a' and 'b' are indicative of unsaturated fatty acids with double bonds in different positions. The positions of these double bonds were not determined in this study.

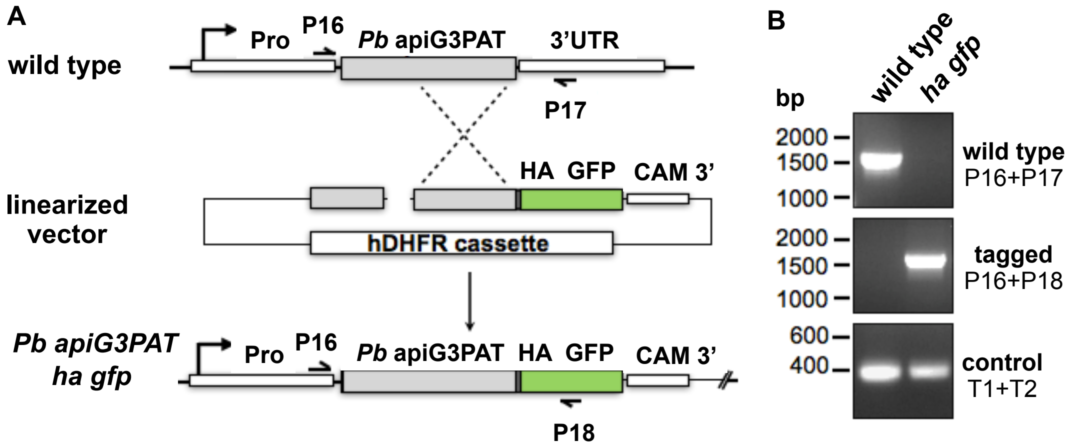

Supplementary Figure 4. *Pb* apiG3PAT tagging strategy and confirmation by PCR. A. Diagram of the strategy used to generate the *Pb apiG3PAT ha gfp* line. Following single crossover homologous recombination, sequences for the hemagglutinin (HA) tag, green fluorescent protein (GFP) and calmodulin 3' untranslated region (CAM 3') are introduced downstream of the *Pb* apiG3PAT coding sequence, enabling expression of the tagged protein from the endogenous promotor. Position of primers used to confirm the integration of the tagging vector are shown, with primers P16+P17 expected to give a 1652 bp product, and primers P16+P18 a 1709 bp product. B. PCR verifies integration of the tagging vector in *Pb apiG3PAT ha gfp* parasites (abbreviated *ha gfp*). PCR products for the wild type parental line shown for comparison.

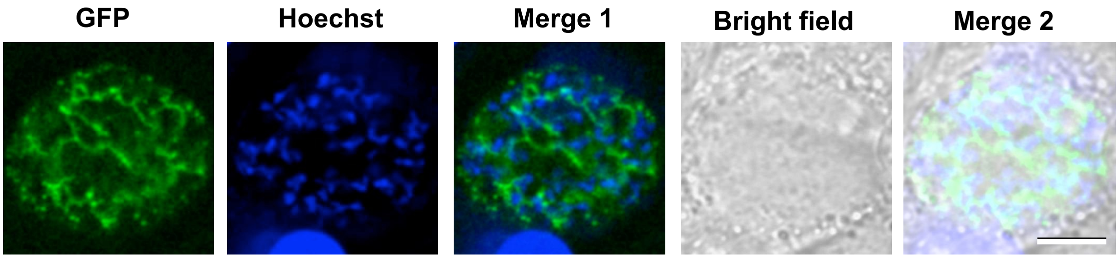

Supplementary Figure 5. Live fluorescence microscopy of *Pb apiG3PAT ha gfp* liver stage parasites. Live fluorescence microscopy of *Pb apiG3PAT ha gfp* liver stage parasites 48 hours post-infection indicates *Pf* apiG3PAT is expressed at this stage and localized in a highly branched structure reminiscent of the apicoplast. DNA stained with Hoechst 33342. Scale 10 µm.

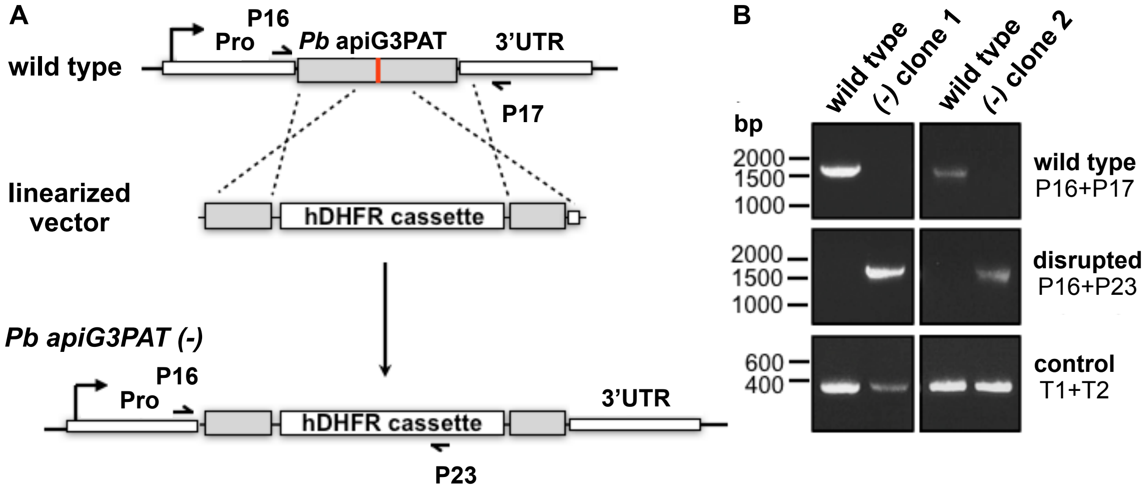

Supplementary Figure 6. *Pb* apiG3PAT knockout strategy and confirmation by PCR. A. Diagram of the strategy used to generate the *Pb apiG3PAT (-)* lines. Following double crossover homologous recombination, the *Pb* apiG3PAT coding sequence is disrupted by the human dihydrofolate reductase (hDHFR) selection cassette, truncating the enzyme and eliminating its putative active site (red). Positions of primers used to confirm disruption of the *Pb* apiG3PAT coding sequence are shown, with primes P16+P17 expected to give a 1736 bp product, and primers P16+P23 a 1736 bp product. B. PCR verifies disruption of the *Pb* apiG3PAT coding sequence in two independent clones (abbreviated *(-)* clone 1 and *(-)* clone 2). PCR products for the wild type parental line shown for comparison.

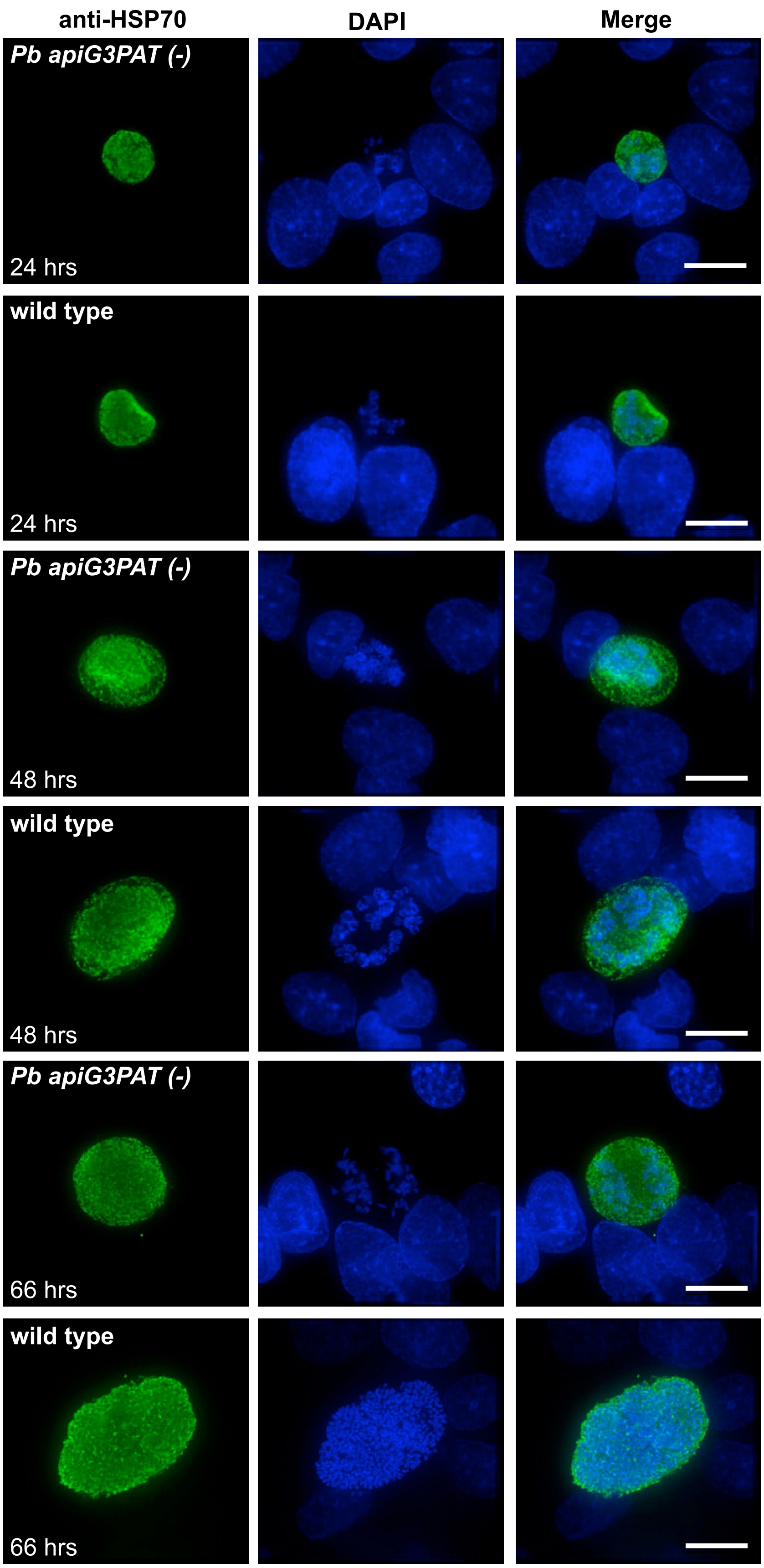

Supplementary Figure 7. Representative immunofluorescence images of *Pb apiG3PAT (-)* and wild type parasites as used for measuring liver stage cell size. Maximum projection images of *Pb apiG3PAT (-)* and wild type parasites labeled with antibodies against the cytosolic marker HSP70 at 24, 48 and 66 hours post-infection. DNA stained with DAPI. Scale 10 µm.

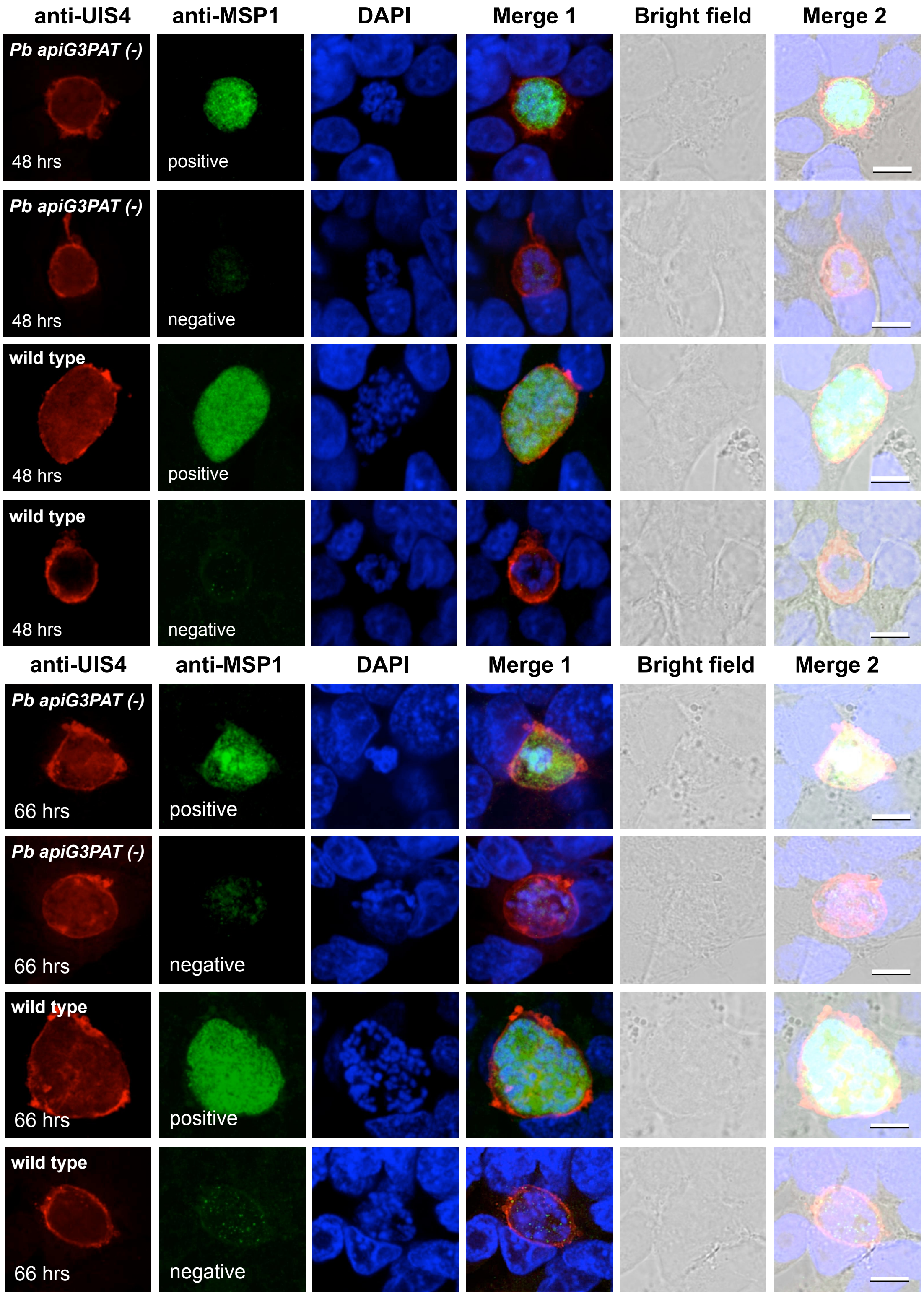

Supplementary Figure 8. Representative immunofluorescence images of *Pb* apiG3PAT *(-)* and wild type parasites as stained for scoring expression of merozoite surface protein 1 (MSP1) in the late liver stage. A. Immunofluorescence images of *Pb apiG3PAT (-)* and wild type parasites using antibodies against upregulated in infectious sporozoites 4 (UIS4) and merozoite surface protein 1 (MSP1) at 48 and 66 hours post-infection. DNA stained with DAPI. Scale 10 μm.
